# Systemic immunosuppression and risk of age-related macular degeneration

**DOI:** 10.1371/journal.pone.0203492

**Published:** 2018-09-20

**Authors:** Harpal S. Sandhu, Joshua Lambert, Yan Xu, Henry J. Kaplan

**Affiliations:** 1 Department of Ophthalmology and Visual Sciences, University of Louisville, Louisville, KY, United States of America; 2 Applied Statistics Lab, University of Kentucky, Louisville, KY, United States of America; Boston University School of Medicine, UNITED STATES

## Abstract

A local immune response has been implicated in the pathogenesis of age-related macular degeneration (AMD), but it is unclear if systemic immunosuppressive/immunomodulatory therapy (IMT) protects against the onset and/or progression of AMD. We performed a retrospective cohort study using a Cox proportional hazards model of two cohorts. Cohort 1 included patients with stage V chronic kidney disease (CKD) status post kidney transplantation, on at least one IMT agent, and older than 50. Cohort 2 included patients with stage IV or V CKD who had not undergone kidney transplantation, were not on IMT, and were older than 50. The main outcomes were hazard ratios of a new diagnosis of dry AMD, wet AMD, or conversion from dry to wet. There were 10,813 patients in cohort 1, and 217,081 patients in cohort 2. After controlling for sex and age, there was no significant difference in the hazard of developing a new diagnosis of dry AMD (HR = 0.95, 95% CI 0.87–1.05, p = 0.32), developing a new diagnosis of wet AMD without any prior diagnosis of dry AMD (HR = 0.85, 95% CI 0.66–1.08, p = 0.18), or converting from dry to wet AMD (HR 1.24, 95% CI 0.94–1.62, p = 0.12). For patients over 70 on mycophenolate mofetil, there was a reduced hazard of converting from dry to wet AMD (HR = 0.92, 95% CI = 0.85–0.99, p = 0.02). In contrast, everolimus had an increased hazard of dry AMD (HR = 2.14, 95% CI 1.24–3.69, p < 0.01). Most systemic IMT does not affect the risk of onset or progression of AMD in patients with CKD. However, mycophenolate mofetil may confer some degree of protection against the conversion of dry AMD to wet AMD, suggesting that modulation of the immune response may prevent progression of the disease.

## Introduction

Age-related macular degeneration (AMD) is the leading cause of vision loss in patients over 65 in the United States [1]. While there have been major advances in treating exudative AMD, long-term outcomes are still poor, with 2/3 of patients experiencing significant vision loss and almost all patients exhibiting some geographic atrophy of the macula after seven years [2,3]. Furthermore, its pathogenesis is still poorly understood. Senescence of the retinal pigment epithelium (RPE) and Bruch’s membrane, oxidative stress, accumulation of metabolic byproducts, and lipid metabolism all appear to play a role, but the exact nature and hierarchy of all these processes are still uncertain[4–10]. In addition, a local immune response has been implicated in the pathogenesis of AMD. Genetic associations between complement factor H and accumulation of drusenoid material beneath Bruch’s membrane are well established [11]. Activated macrophages also play a role in the pathogenesis of choroidal neovascularization. Moreover, there is a well-established association between mutated high temperature serine protease (HTRA-1) and AMD [12].This protease is thought to regulate TGF-beta and cleaves fibronectin, whose fragments stimulate proinflammatory cytokines and matrix metalloproteinases. Fas ligand (FasL), a key downregulator of the immune response in the eye, is important in inhibiting pathological angiogenesis in the RPE [13]. In a similar vein, systemic immunosuppression in animal models has been shown to inhibit choroidal neovascularization [14].

While corticosteroid therapy in AMD has shown little efficacy [15, 16, 17], a small pilot study at the National Eye Institute investigating concomitant use of systemic immunosuppressive/immunomodulatory therapy (IMT) showed initial promise in decreasing the frequency of anti-VEGF injections in patients with pre-existing exudative AMD [18]. Since then, no larger trials of systemic IMT have been conducted, and trials modulating the complement cascade have failed to halt the growth of geographic atrophy [19]. Of course, there are many patients on systemic IMT for diseases not involving the eye. This begs the following questions: do they have a lower incidence of AMD? If such patients develop dry AMD while on IMT, do they convert to wet AMD at a lower rate?

The purpose of this study was to determine if systemic IMT protected against the onset and/or progression of AMD. Immunomodulatory therapy is broadly indicated for two groups of patients: those with systemic inflammatory/autoimmune diseases and those with organ allografts. The former group presents major problems in this endeavor because of confounding by indication. The very reason for their being prescribed IMT, namely a systemic inflammatory disease, establishes an aberrant immune response and may increase the risk or accelerate the progression of AMD. This question remains open, but sufficient evidence exists to entertain the hypothesis. For instance, patients with psoriasis, a systemic inflammatory disease characterized by a T cell response against cutaneous antigens, have been shown to have a higher risk of AMD [20]. Myeloproliferative disorders, hematologic neoplasms associated with chronic inflammation, have also been linked to AMD [21]. This leaves for study the population of patients who are on IMT to suppress allograft rejection after organ transplantation. The chronic kidney disease (CKD) population is a suitable group to study for two reasons. First, advanced CKD can be treated by transplantation plus IMT or by non-transplantation means, allowing for a natural comparison group with a similar underlying disease severity. Second, the kidney is the most commonly transplanted organ in the United States and worldwide. In the United States, approximately 17,000 renal allografts are performed each year [22], and all these patients must be on lifelong IMT, typically starting with three agents and eventually weaning down to one or two. This study sought to compare the incidence of dry and wet AMD in patients on IMT because of advanced CKD status post renal transplantation to patients with advanced CKD but without a transplant, and thus not on IMT. For patients with a diagnosis of dry AMD, we sought to compare rates of conversion to wet AMD between those on IMT and those who were not.

## Methods

### Dataset

The Truven Analytics (Fort Worth, TX, USA) dataset includes approximately 80 million patients enrolled in multiple different private health insurance plans across the United States from 2010 to 2015. It includes de-identified demographic information, all ICD-9 diagnosis codes from all outpatient visits and inpatient hospitalizations, records of all prescribed medication, and all procedural codes. Because of the de-identified nature of the data, it was exempt from review by the University of Louisville’s institutional review board.

### Study design

This was a retrospective cohort study. Cohort one was the IMT cohort. Inclusion criteria included age greater than 50, a diagnosis of stage V CKD, commonly referred to as end-stage renal disease (ESRD), a procedure code for renal transplantation, and a prescription for at least one of eight IMT agents or oral prednisone. Cohort two was the control or non-IMT group. Inclusion criteria included age greater than 50 and a diagnosis of stage IV or V CKD. Exclusion criteria for both groups were a diagnosis of a common systemic inflammatory, autoimmune, or rheumatic disease, a diagnosis of membranoproliferative glomerulonephritis, or a diagnosis of an inherited retinal degeneration (IRD) (Table 1). Mebranoproliferative glomerulonephritis was excluded because of its close association with a form of macular degeneration. Inherited degenerations were excluded because of the diagnostic confusion that would ensue if a patient had a diagnosis code for an IRD but was then later also given a diagnosis of AMD. The index date for cohort one was the date of the first recorded prescription of any IMT agent. For cohort two, it was the first date listing the diagnosis of stage IV or V CKD. Patients were censored for a diagnosis of any form of AMD, death, or departure from the dataset. An initial six-month period without any diagnosis of AMD was required. The purpose of such a lead-in period was to capture only new or incident cases of AMD. Outcomes of interest were the hazard ratio of developing a new diagnosis of dry AMD, a diagnosis of wet AMD without a prior diagnosis of dry, and conversion from a diagnosis of dry AMD to wet AMD.

**Table 1:** Diagnoses and corresponding ICD9 codes excluded from this study.

Diagnosis	ICD9 code
***Rheumatic Diseases***	
Connective tissue diseases	710.x
Rheumatoid arthritis and rheumatoid arthritis variants	714.x
Behcet's disease	136.1
Anklyosing spondylitis	720.0
Psoriatic arthritis	696.0
Systemic vasculitides	446.x
***Dermatologic Diseases***	
Psoriasis	696.x
***Neurological Diseases***	
Multiple sclerosis	340
Other demyelinating disorder	341.x
***Gut Diseases***	
Crohn's disease	555.x
Idiopathic proctocolitis, ulcerative colitis	556.x
Primary biliary cirrhosis, autoimmune hepatitis	571.x
***Hematopoietic Diseases***	
Chronic myelogenous leukemia	205.1
Myeloproliferative disorders	238.x
***Renal Disease***	
Membranoproliferative glomerulonephritis	581.2
***Retinal Diseases***	
Hereditary retinal dystrophies	362.7x
***Other***	
Sarcoidosis	135.0

### Statistical analysis

A multivariate Cox proportional hazards model was employed to determine the hazard of a new diagnosis of dry AMD, wet AMD, and conversion from dry to wet. All hazards were expressed as the ratio of the hazard in cohort 1 to the hazard in cohort 2. Sex and age were used as covariates in order to control for the key role that advanced age plays in the disease. Data on race and cigarette smoking were not in the dataset and thus could not be covariates. All statistical analysis was performed using Stata (College Station, TX, USA).

## Results

**Fig 1:**
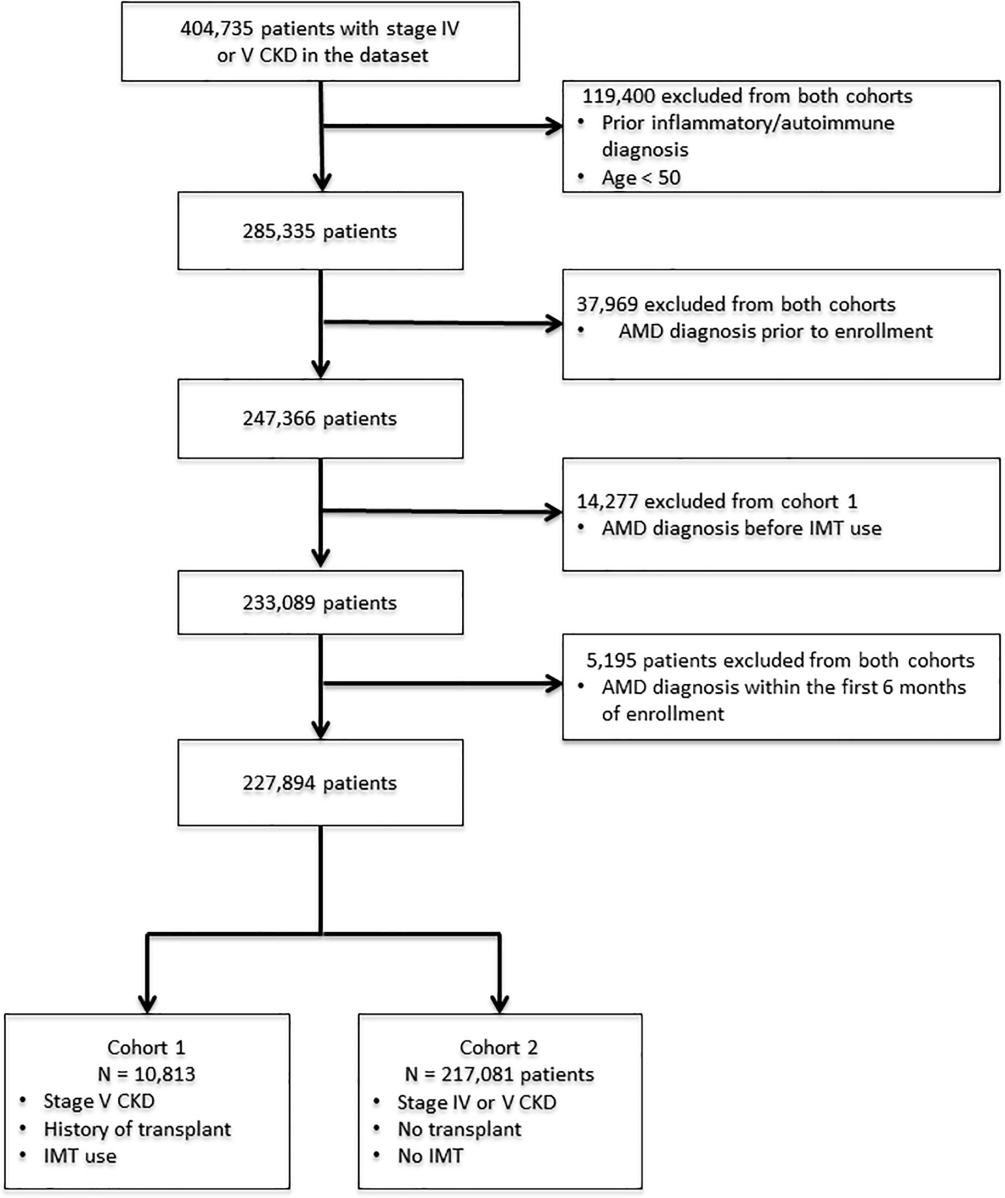
Flow chart of patients in each cohort after applying exclusion and inclusion criteria.

There were 404,735 patients in the dataset with stage IV or V CKD. After inclusion and exclusion criteria were applied, there were 10,813 patients in cohort 1 and 217,081 in cohort 2 (Fig 1). Cohort 1 was 36.6% female and cohort 2 44.6%. Average age in cohort 1 was 59.4 years (interquartile range [IQR] 9). Average age in cohort 2 was 71.5 years (IQR 19) (Table 2).

**Table 2:** Baseline characteristics of the study population by cohort.

	Cohort 1	Cohort 2
Number of patients	10,813	217,081
Mean age (IQR)	59.4 years (9)	71.5 years (19)
% Female	36.6%	44.6%

469 patients (4.3%) in cohort 1 and 8123 patients (3.7%) in cohort 2 developed a diagnosis of dry AMD. By univariate analysis, the hazard ratio of developing dry AMD in cohort 1 relative to cohort 2 was 0.98 (95% CI 0.90–1.08, p = 0.76). When controlling for age in multivariate analysis, the difference in hazard was still negligible (HR = 0.95, 95% CI 0.87–1.05, p = 0.32) (Table 3).

**Table 3:** Hazard ratios of developing dry AMD, wet AMD without a prior diagnosis of dry, or conversion from dry to wet.

		Hazard Ratio (HR Cohort 1/HR cohort 2)	95% CI	P value
Dry	Univariate	0.98	0.90–1.08	0.76
	Multivariate	0.95	0.87–1.05	0.32
Wet	Univariate	0.87	0.69–1.11	0.26
	Multivariate	0.85	0.66–1.08	0.18
Dry to Wet	Univariate	1.17	0.91–1.52	0.23
	Multivariate	1.24	0.94–1.62	0.12

The wet AMD outcome is actually two distinct outcomes. The first is those patients who are diagnosed with wet AMD without a prior diagnosis code for dry AMD. The second is those patients who harbor an existing diagnosis of dry AMD and then later are diagnosed with wet AMD, which we call conversion from dry to wet. With regard to the former group, 70 patients in cohort 1 (0.64%) and 1435 patients (0.66%) in cohort 2 developed a diagnosis of wet AMD without any prior diagnosis of dry. By univariate analysis, the hazard ratio of developing wet AMD in cohort 1 relative to cohort 2 was 0.87 (95% CI 0.69–1.11, p = 0.26). Again, in multivariate analysis, this effect was still not significant (HR = 0.85, 95% CI 0.66–1.08, p = 0.18) (Table 3).

For conversion from dry to wet AMD, in both univariate and multivariate analysis, there was no statistically significant difference in hazard between the two cohorts (univariate: HR 1.17, 95% CI 0.91–1.52, p = 0.23; multivariate HR 1.24, 95% CI 0.94–1.62, p = 0.12) (Table 3).

Patients were then divided into subgroups based on a prescription for individual IMT agents and analyzed for the same outcomes. Nine different agents were examined: the corticosteroid prednisone; the anti-metabolites methotrexate, azathioprine, and mycophenolate mofetil; the calcineurin inhibitors cyclosporine and tacrolimus; the mechanistic/mammalian target of rapamycin (mTOR) inhibitors sirolimus and everolimus; and the T cell co-stimulation antagonist belatacept (soluble receptor for the ligand B7 on antigen-presenting cells). The vast majority of prescriptions were for prednisone, mycophenolate mofetil, and tacrolimus (Table 4), which together constitute the most common triple immunomodulatory regimen for renal allograft patients. One agent, everolimus, had an increased hazard of developing dry AMD (HR = 2.14, 95% CI 1.24–3.69, p < 0.01) (Table 4). Of note, only 132 patients were on the agent, and 10 (7.6%) developed dry AMD. In the wet AMD group, there was no statistically significant change in hazard for any agent. When patients who converted from dry to wet AMD were analyzed by IMT drug, mycophenolate mofetil showed a reduction in hazard (HR = 0.92, 95% CI = 0.85–0.99, p = 0.02) in older patients, namely those over 70. However, across all ages, mycophenolate showed no difference in hazard (HR 1.03, 95% CI 0.69–1.52, p = 0.90, Table 4). No other drug showed any effect in conversion from dry to wet AMD. All potential combinations of two or three drugs were also examined. No pair or trio of drugs had any statistically significant effect.

**Table 4:** Hazard ratios of developing dry AMD, wet AMD without prior diagnosis of dry, or conversion from dry to wet by IMT agent in cohort 1 patients. Results significant at the p = 0.05 level are in bold.

	Total number of patients with a prescription	Hazard Ratios		
		Dry AMD (95% CI, p value)	Wet AMD (95% CI, p value)	Conversion from Dry to Wet AMD (95% CI, p value)
Mycophenolate mofetil	5539	0.92 (0.81–1.05, p = 0.23)	1.06 (0.79–1.43, p = 0.70)	1.03 (0.69–1.52, p = 0.90)
Azathioprine	341	0.82 (0.48–1.38, p = 0.44)	1.03 (0.33–3.19, p = 0.96)	0.56 (0.08–3.97, p = 0.56)
Methotrexate	3	0.0001 (0-infinitiy, p = 0.96)	0.0001 (o-infinity, p = 0.98)	0.0001 (0-infinity, p = 0.985)
Tacrolimus	6128	0.92 (0.81–1.04, p = 0.17)	0.92 (0.68–1.25, p = 0.60)	1.25 (0.89–1.76, p = 0.202)
Sirolimus	147	0.86 (0.39–1.92, p = 0.72)	0.87 (0.12–6.16, p = 0.89)	1.42 (0.20–10.1, p = 0.724)
Everolimus	126	**2.14 (1.24–3.69, p = 0.006)**	2.10 (0.52–8.43, p = 0.29)	0 (0-infinity, p = 0.98)
Cyclosporine	871	1.07 (0.79–1.44, p = 0.68)	1.03 (0.49–2.18, p = 0.93)	0.74 (0.24–2.30, p = 0.60)
Belatacept	5	2.75 (0.39–19.5, p = 0.31)	0.0001 (0-infinitiy, p = 0.98)	0.0001 (0-infinity, p = 0.99)
Prednisone	8153	0.94 (0.84–1.04, p = 0.23)	0.83 (0.63–1.11, p = 0.21)	1.24 (0.92–1.68, p = 0.16)

## Discussion

Human histopathology and animal studies of AMD have consistently demonstrated a local immune response in this disease. What has yet to be demonstrated is that modulation of this immune response can have a therapeutic effect. In this study, most IMT in the CKD population had no effect, either helpful or harmful, on the risk of onset or progression of AMD. However, one agent, mycophenolate mofetil, showed a modest but statistically significant reduction in the hazard of converting from dry AMD to wet AMD, and one agent, everolimus, had an increased hazard of dry AMD.

The immune response in AMD is complex and incompletely understood. Multiple molecules and cells across both the innate and adaptive immune systems are involved, but the hierarchy and importance of such mechanisms in the broader context of retinal, RPE, and Bruch’s membrane senescence have yet to be established. Drusen themselves consist partly of complement factors, and polymorphisms in complement factor H, a downregulator of complement, are a well-known risk factor for AMD. When certain complement components are blocked in the laser-induced model of CNV, neovascularization does not develop [23]. In addition, a monocytic infiltrate is present in AMD [24, 25], and alternatively activated macrophages (M2 macrophages) are known contributors to angiogenesis. Alternatively activated macrophages do not exhibit the pro-inflammatory phenotype of classically activated ones. Instead, they express TGF-beta and Il-10, contribute to wound healing, and induce angiogenesis [26]. In multiple studies of macrophage depletion in mice, loss of macrophages inhibits choroidal neovascularization [27, 28]. We should add that the appropriate cytokine milieu, mostly involving Il-10 [29], is necessary to polarize macrophages towards the pro-angiogenic M2 subtype, or else the anti-angiogenic, M1 subtype dominates [30]. Indeed, Il-10 increases in the aging eye [31], polarizing them to the M2 subtype. Furthermore, loss of FasL causes increased choroidal neovascularization in mice [13]. These findings have led to the invocation of a “response to retention” hypothesis in AMD pathogenesis, not unlike atherosclerosis [32]. Due to RPE senescence, lipoproteins begin to accumulate beneath the RPE. These retained products, some of which are oxidized, elicit a subclinical immune response, hence the phrase response to retention. This includes macrophages that, when alternatively activated, degrade Bruch’s membrane with matrix metaloproteinases and ultimately can induce angiogenesis. This shares certain commonalities with atherosclerosis, in which oxidized lipoproteins are retained in a subintimal location, inciting a chronic immune response rich in macrophages that increases vascular plaque build up [33]. One can also broadly conceive of both pathologies as wound healing responses to abnormal or damaged tissue. Multiple studies have shown cardiovascular benefit from chronic IMT [34–38], and interestingly canakinumab, an Il-1 beta inhibitor, reduced mortality from coronary artery disease in a recent randomized controlled trial [39].

Mycophenolate mofetil has multiple mechanisms of action, at least one of which may explain the protective effect found herein. As an inosine monophosphate (IMP) dehydrogenase inhibitor, it primarily antagonizes lymphocytes. Both T and B lymphocytes cannot produce guanosine de novo and must rely on the salvage pathway in order to replicate DNA. Because IMP dehydrogenase is essential to the salvage pathway but not the de novo one, mycophenolate is relatively selective for lymphocytes. T and B cells are largely absent from the immune response in AMD, and thus this mechanism is unlikely to be the causative one. On the other hand, by reducing glycosylation of intercellular and vascular cell adhesion molecules, mycophenolate reduces the infiltration of pro-angiogenic M2 macrophages and monocytes into the eye. Bone marrow-derived stem cells are also recruited via vascular adhesion molecules and are increasingly recognized as important in angiogenesis. They differentiate into endothelial progenitor cells and incorporate into the nascent choroidal neovascular membrane [40, 41, 42]. Because mycophenolate prevents these cells from leaving the bloodstream and entering the choroid and RPE, their potential to stimulate or sustain choroidal neovascularization would theoretically be inhibited.

The canonical inflammasome also plays a role in the pathogenesis of advanced AMD, and mycophenolate’s downregulatory effect on Il-1 beta is a third biologically plausible mechanism by which the drug could protect against conversion from dry to wet AMD. The inflammasome is a complex of pro-Il-1 beta and Il-18 that becomes active and released from inflammatory cells after cleavage by caspase-1, which in turn is activated by signals from NOD-like receptor family pyrin domain 3 (NLRP3). Excessive inflammasome activity has been implicated in other neurodegenerative diseases such as Alzheimer’s [43]. While Il-18’s role in advanced AMD is unclear–there is mixed evidence that it might inhibit CNV and some that it may promote geographic atrophy [44–47], Il-1 beta is clearly a pro-angiogenic cytokine. The drusen component A2E induces Il-1 beta expression [48], which promotes angiogenesis both in a VEGF-dependent and VEGF-independent fashion [49, 50, 51]. Moreover, inhibition of Il-1 beta has decreased the size of CNV lesions in preclinical studies. Mycophenolate downregulates expression of Il-1 beta by leukocytes and microglia, which would theoretically decrease choroidal neovascularization [52, 53, 54]. As mentioned, there is now a selective Il-1 beta inhibitor which has shown a mortality benefit in cardiovascular disease, and these data as well as the notable similarities between the two diseases raise the prospect of Il-1 beta modulation as therapy for AMD.

The drug’s last mechanism of action, inhibition of inducible nitric oxide synthase, is less likely to explain any therapeutic effect. While nitric oxide can cause oxidative damage to the RPE, evidence for a specific role in AMD pathogenesis is limited.

A second finding in this study was the harmful effect of everolimus, which increased the hazard of developing dry AMD by over two fold. Everolimus is an mTOR inhibitor. The mechanistic/mammalian target of rapamycin is a serine-threonine protease associated with a large protein complex that integrates various signals from growth factors and cellular nutrients to direct protein synthesis, cell growth, and cell proliferation. It is also integral to allowing lymphocytes to enter the S phase of the cell cycle, and thus is effectively a T cell inhibitor. As explained above, lymphocytes are rarely present in AMD histopathology, and thus one would not intuitively expect T cell inhibition to be therapeutic in this disease. This study supports such a rationale, as no T cell inhibitor showed a protective effect. However, observing a harmful effect raises questions. A small, phase I study of intravitreal sirolimus, another mTOR inhibitor, for geographic atrophy (GA) in AMD was halted early because two of the six patients developed rapid, progressive central retinal thinning on OCT [55]. The investigators noted that mTOR affects multiple intracellular pathways, rendering the ultimate effect of its inhibition unpredictable. Some preclinical studies have shown a beneficial effect from mTOR inhibition on RPE senescence while others have shown that mTOR physiologically extends photoreceptor survival in animal models of retinal degeneration and mediates the RPE’s response to nerve growth factors. The exact reason for everolimus’ harmful effect in this study is unclear. As is the case for any of the effects seen in this study, one must consider the possibility that an off-target drug effect is responsible. Last, this is a single study whose results require further validation, and thus there remains the possibility that these effects are spurious.

This study has several notable limitations. First, it did not control for smoking or race, both risk factors for AMD to different extents. Smoking is not coded for by ICD9 or 10 diagnoses, and race was not included in the dataset, thus making it impossible to control for either.

Second, both incident and prevalent cases of kidney transplants were included in this study, which has both benefits and drawbacks. Most renal allograft recipients in the US are middle aged at the time of transplantation while onset of AMD correlates more strongly with age than any other risk factor. Including only incident cases of renal transplant recipients over 50 would greatly reduce the sample size, hazard of disease, and power of the study. Conversely, by including prevalent transplant cases with older patients (i.e. patients who underwent transplantation prior to their entering the insurance dataset), the sample, hazard of disease, and power of the study all increase. The drawback to this approach is that the IMT medication history of these patients cannot be tracked prior to their entering the dataset. While all allograft recipients must be on some continuous IMT, we do not know the exact nature of said regimen for each patient. For example, a patient in this study on mycophenolate alone for five years may have always been on mycophenolate, or she may have been switched from azathioprine to mycophenolate a few years prior to inclusion in this dataset. Because the prior regimen is unknown, the extent to which other agents may have contributed to the protective effect seen for mycophenolate is unclear. Having stated that, because no other agents showed a protective effect and one even showed a deleterious one, it is more likely that these prior unknowns have diluted mycophenolate’s protective effect rather than enhanced it. Moreover, we posit that the beneficial interaction effect seen between patient age and mycophenolate use is probably a surrogate for length of use of mycophenolate, the standard of care anti-metabolite agent for allograft recipients since its FDA approval in 2000. Thus, the effect of mycophenolate is strongest in the very group of patients who are at higher risk of developing the disease due to their advanced age. Similarly, by the same logic, the true harmful effect of everolimus may also be diluted. Since most renal transplants occur in middle age but AMD’s onset is largely in the elderly, only a dataset with 20–30 years of continuous patient data can address this issue rigorously.

Third, although it is clear that immune dysregulation has a role in the development of CNV, and possibly dry AMD, the uremic state is associated with disorders of the innate and adaptive immune systems in ESRD [56]. Thus, a state of acquired immune dysfunction in uremia could be an important confounding variable for both the development of dry and wet AMD. Until a better understanding of the pathophysiology of AMD is obtained the importance of such changes in the population studied are unknown.

Finally, as in all retrospective cohort studies, even when employing multiple covariates and matching for kidney disease severity, it is always possible that some unidentified, causal risk factor does not segregate evenly between groups, thereby introducing systematic bias.

The strengths of this study include its large sample size, its use of a transplant population to mitigate confounding by indication (indication bias), and its novelty. To the best of our knowledge, this is the first study to examine systemic IMT for AMD in a large set of patients. Using the advanced CKD population substantially reduces confounding, a fatal flaw in many studies of similar methodology, because either dialysis or transplantation plus IMT is a viable option for advanced CKD as described in the introduction.

More research is needed to corroborate these results both in other well designed studies of different organ allografts and perhaps also in patients with chronic inflammatory diseases who were deliberately excluded from this study because of the notable caveats listed above. Moreover, while the mTOR inhibitors have been the subject of considerable pre-clinical work in this area, the same is untrue of anti-metabolites or other IMT agents. A logical next step would be to test these agents for efficacy in animal models of AMD and choroidal neovascularization. It would also be intriguing to see if everolimus recapitulates or accelerates an AMD phenotype in susceptible animal models. While systemic IMT carries considerable systemic health risks, one hopes local IMT agents might equal or even surpass systemic ones in efficacy for this blinding disease.

## Conclusion

In general, most systemic IMT does not affect the risk of onset or progression of AMD in patients with chronic kidney disease. However, mycophenolate mofetil may confer some degree of protection against the conversion of dry AMD to wet AMD, suggesting that modulation of the immune response may prevent progression of the disease. Conversely, everolimus may increase the risk of dry AMD. More research is needed to validate these findings in other patient populations. If such validation comes to fruition, more work would be needed to elucidate the exact immunologic mechanisms by which these effects occur.

## Supporting information

S1 File Second short report. This document summarizes baseline numbers of each subgroup after application of inclusion and exclusion criteria and every analysis performed.
(PDF)
