# Supplementary material for: Systemic immunosuppression and risk of age-related macular degeneration
Source: PLoS One. 2018 Sep 20;13(9):e0203492. doi: 10.1371/journal.pone.0203492 (PMC6147423; doi:10.1371/journal.pone.0203492)
Supplement: S1 File — This document summarizes baseline numbers of each subgroup after application of inclusion and exclusion criteria and every analysis performed. (PDF) [file pone.0203492.s001.pdf]

Analysis for Dry AMD, Wet AMD and NOS Diagnosis

Joshua Lambert, PhD and Yan Xu; University of Kentucky Department of Statistics

Feb 13, 2018

Dry AMD

For the dry amd group a basic table of sex, age, death, censor and survival time are displayed. And for each specific drug, tables are listed broken down by basic demographics. Cox proportional hazards regression model is used to assess the effects of age and each drug. The main significant finding here is that minimum age and drug everolimus are significant. These results suggest that those who were older had a smaller hazard of developing Dry AMD (Hazard Estimate: 0.998, 95% CI:(0.9956,0.9993), p-value = 0.0063) for every year older they were. Also, these results suggest that after adjusting for miniumum age, those who were on everolimus in the Kidney Transplant cohort group had an increased hazard (Hazard Estimate: 2.14, 95% CI: (1.24,3.69), p-value=0.0061) compared to those who were not on everolimus in the non Kidney Transplant group for developing dry AMD. Also, a statistical interaction between sex and mycophenolate mofetil was found (HR Estimate=1.37, 95% CI: (1.0561,1.7805), p-value=0.0178). This interaction suggests that females on the drug have a higher estimated hazard for developing Dry AMD than females not on the drug (adjusted for age) while men on the drug have a smaller estimated hazard for developing Dry AMD than males not on the drug (adjusted for age).

Simple Dry AMD Tables

Here a a list of simple tables related to Dry AMD.

```
res_tab_dry #breakdown of dry amd. 1=Had Dry AMD only NA=Did not have Dry AMD
```

```
     1   NA's    Sum
  8592 215956 224548
```

```
sex_tab_dry #breakdown of dry amd by sex. 1=Women 2=Men
```

```
     1      2    Sum
125283  99265 224548
```

```
plot(age_hist_dry) #breakdown of dry amd: histogram of minimum age for each subject
```

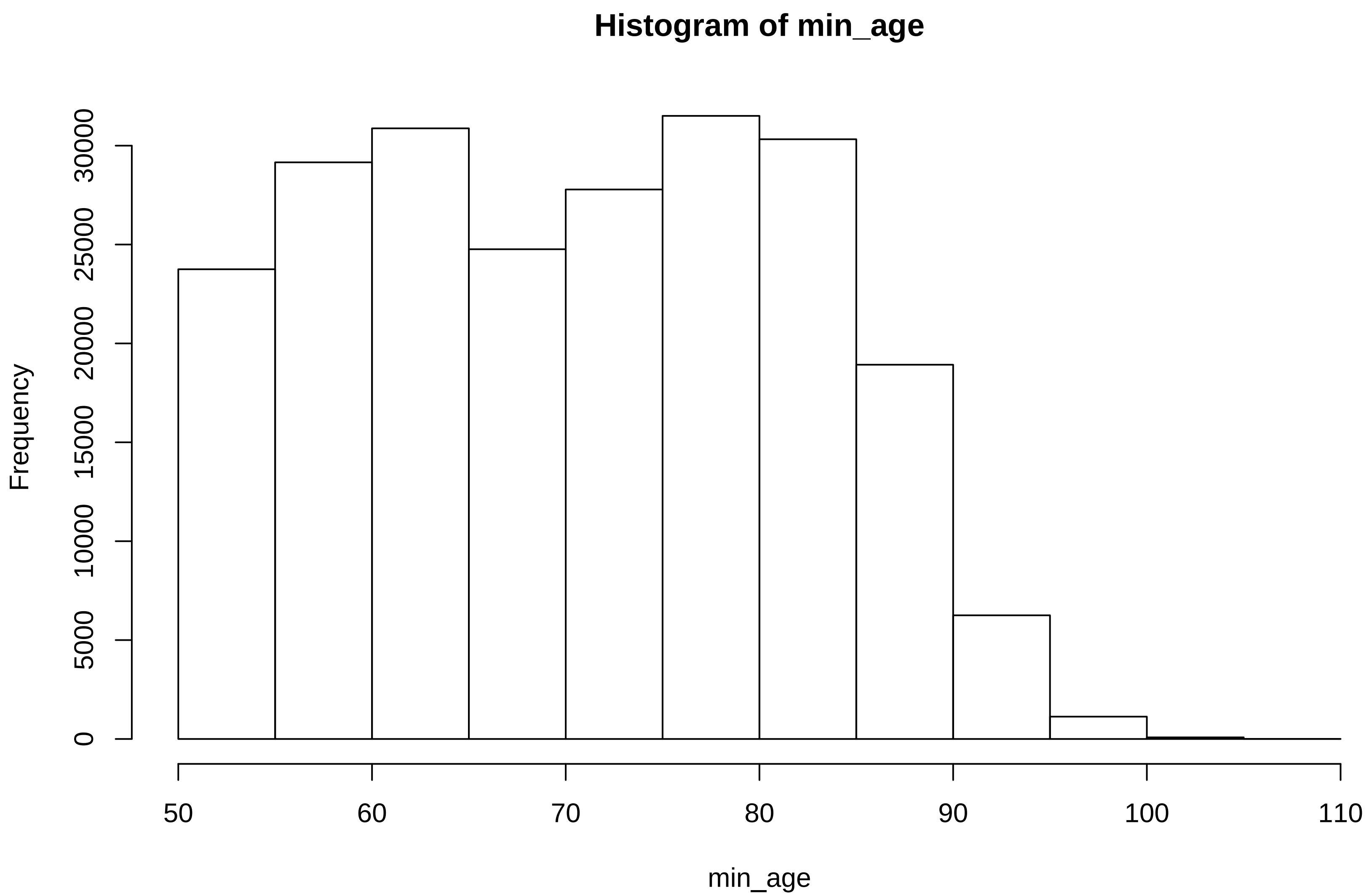


```
age_tab_dry #breakdown of dry amd: summary table of miniumum age
```

```
   Min. 1st Qu.  Median    Mean 3rd Qu.    Max. 
  50.00   61.00   71.00   70.94   81.00  107.00 
```

```
death_tab_dry #breakdown of dry amd: 1=Died; NA=No infomation about death
```

```
     1   NA's    Sum 
 11883 212665 224548 
```

```
censor_tab_dry #breakdown of dry amd: 1=Censored; 0=No Censored
```

```
     0      1    Sum 
  8592 215956 224548 
```

```
survmon_tab_dry #breakdown of dry amd: summary table of the survival months by subject
```

```
   Min. 1st Qu.  Median    Mean 3rd Qu.    Max. 
  0.000   4.046  11.000  15.997  23.750  60.033 
```

There were 8592 subjects who had dry AMD and were in cohort 1.

Dry AMD Tables by Drug

Here a a list of ables related to Dry AMD by drug.

```
#top part of tables are for those who were not on the drug (0)
#bottom part of tables are for those who were on the drug (1)
mycomo_bin_tab_dry # mycophenolate mofetil
```

```
dry_data[-which(dry_data$group == "c1" & dry_data[, "mycomo_bin"] == : 0
 dryamdonly       min_age        sex          death            survmon      
 1   :  8123   Min.   : 50.00   1:118541   1    : 11257   Min.   : 0.000  
 NA's:205793   1st Qu.: 62.00   2: 95375   NA's:202659   1st Qu.: 4.013  
               Median : 72.00                            Median :10.954  
               Mean   : 71.51                            Mean   :15.844  
               3rd Qu.: 81.00                            3rd Qu.:23.605  
               Max.   :107.00                            Max.   :60.033  
--------------------------------------------------------- 
dry_data[-which(dry_data$group == "c1" & dry_data[, "mycomo_bin"] == : 1
 dryamdonly     min_age        sex       death          survmon      
 1   : 238   Min.   :50.00   1:3571   1    : 338   Min.   : 0.000  
 NA's:5301   1st Qu.:54.00   2:1968   NA's:5201   1st Qu.: 4.967  
             Median :58.00                         Median :13.980  
```

```
             Mean   :59.15                     Mean   :19.166
             3rd Qu.:63.00                     3rd Qu.:29.178
             Max.   :87.00                     Max.   :60.000
```

```r
metho_bin_tab_dry #methotrexate
```

```
dry_data[-which(dry_data$group == "c1" & dry_data[, "metho_bin"] == : 0
 dryamdonly       min_age       sex         death           survmon
 1   :  8123   Min.   : 50.00   1:118541   1   : 11257   Min.   : 0.000
 NA's:205793   1st Qu.: 62.00   2: 95375   NA's:202659   1st Qu.: 4.013
               Median : 72.00                            Median :10.954
               Mean   : 71.51                            Mean   :15.844
               3rd Qu.: 81.00                            3rd Qu.:23.605
               Max.   :107.00                            Max.   :60.033
--------------------------------------------------------
dry_data[-which(dry_data$group == "c1" & dry_data[, "metho_bin"] == : 1
 dryamdonly    min_age    sex    death      survmon
 1   :0     Min.   :52   1:2   1   :0   Min.   : 7.375
 NA's:3     1st Qu.:54   2:1   NA's:3   1st Qu.:30.529
            Median :56                  Median :53.684
            Mean   :62                  Mean   :40.353
            3rd Qu.:67                  3rd Qu.:56.842
            Max.   :78                  Max.   :60.000
```

```r
cyclo_bin_tab_dry #cyclosporine
```

```
dry_data[-which(dry_data$group == "c1" & dry_data[, "cyclo_bin"] == : 0
 dryamdonly       min_age       sex         death           survmon
 1   :  8123   Min.   : 50.00   1:118541   1   : 11257   Min.   : 0.000
 NA's:205793   1st Qu.: 62.00   2: 95375   NA's:202659   1st Qu.: 4.013
               Median : 72.00                            Median :10.954
               Mean   : 71.51                            Mean   :15.844
               3rd Qu.: 81.00                            3rd Qu.:23.605
               Max.   :107.00                            Max.   :60.033
--------------------------------------------------------
dry_data[-which(dry_data$group == "c1" & dry_data[, "cyclo_bin"] == : 1
 dryamdonly    min_age      sex      death        survmon
 1   : 43   Min.   :50.00   1:545   1   : 58   Min.   : 0.00
 NA's:828   1st Qu.:55.00   2:326   NA's:813   1st Qu.: 4.95
            Median :59.00                      Median :14.24
```

```
           Mean   :59.94                      Mean   :19.08
           3rd Qu.:63.00                      3rd Qu.:28.09
           Max.   :86.00                      Max.   :60.00
```

```
tacro_bin_tab_dry #tacrolimus
```

```
dry_data[-which(dry_data$group == "c1" & dry_data[, "tacro_bin"] == : 0
 dryamdonly      min_age        sex           death             survmon
 1   :  8123   Min.   : 50.00   1:118541   1   : 11257   Min.   : 0.000
 NA's:205793   1st Qu.: 62.00   2: 95375   NA's:202659   1st Qu.: 4.013
               Median : 72.00                            Median :10.954
               Mean   : 71.51                            Mean   :15.844
               3rd Qu.: 81.00                            3rd Qu.:23.605
               Max.   :107.00                            Max.   :60.033
--------------------------------------------------------
dry_data[-which(dry_data$group == "c1" & dry_data[, "tacro_bin"] == : 1
 dryamdonly     min_age       sex        death          survmon
 1   : 260   Min.   :50.00   1:3889   1   : 381   Min.   : 0.000
 NA's:5868   1st Qu.:54.00   2:2239   NA's:5747   1st Qu.: 4.946
             Median :58.00                        Median :13.819
             Mean   :58.91                        Mean   :19.054
             3rd Qu.:62.00                        3rd Qu.:29.416
             Max.   :89.00                        Max.   :60.000
```

```
siro_bin_tab_dry #sirolimus
```

```
dry_data[-which(dry_data$group == "c1" & dry_data[, "siro_bin"] == : 0
 dryamdonly      min_age        sex           death             survmon
 1   :  8123   Min.   : 50.00   1:118541   1   : 11257   Min.   : 0.000
 NA's:205793   1st Qu.: 62.00   2: 95375   NA's:202659   1st Qu.: 4.013
               Median : 72.00                            Median :10.954
               Mean   : 71.51                            Mean   :15.844
               3rd Qu.: 81.00                            3rd Qu.:23.605
               Max.   :107.00                            Max.   :60.033
--------------------------------------------------------
dry_data[-which(dry_data$group == "c1" & dry_data[, "siro_bin"] == : 1
 dryamdonly    min_age       sex     death         survmon
 1   :  6    Min.   :50.00   1:97   1   : 12   Min.   : 0.000
 NA's:141    1st Qu.:52.50   2:50   NA's:135   1st Qu.: 4.838
             Median :57.00                     Median :14.046
```

```
           Mean   :57.69                     Mean   :19.470
           3rd Qu.:61.00                     3rd Qu.:30.115
           Max.   :76.00                     Max.   :60.000
```

```
azath_bin_tab_dry #azathioprine
```

```
dry_data[-which(dry_data$group == "c1" & dry_data[, "azath_bin"] == : 0
 dryamdonly       min_age       sex          death           survmon
 1    :  8123   Min.   : 50.00   1:118541   1    : 11257   Min.   : 0.000
 NA's:205793   1st Qu.: 62.00   2: 95375   NA's:202659   1st Qu.: 4.013
                Median : 72.00                             Median :10.954
                Mean   : 71.51                             Mean   :15.844
                3rd Qu.: 81.00                             3rd Qu.:23.605
                Max.   :107.00                             Max.   :60.033
--------------------------------------------------------
dry_data[-which(dry_data$group == "c1" & dry_data[, "azath_bin"] == : 1
 dryamdonly    min_age      sex      death        survmon
 1    : 14    Min.   :50.00   1:186   1    : 21    Min.   : 0.000
 NA's:327    1st Qu.:53.00   2:155   NA's:320    1st Qu.: 7.105
             Median :57.00                        Median :17.204
             Mean   :58.38                        Mean   :20.761
             3rd Qu.:62.00                        3rd Qu.:31.980
             Max.   :83.00                        Max.   :59.967
```

```
evero_bin_tab_dry #everolimus
```

```
dry_data[-which(dry_data$group == "c1" & dry_data[, "evero_bin"] == : 0
 dryamdonly       min_age       sex          death           survmon
 1    :  8123   Min.   : 50.00   1:118541   1    : 11257   Min.   : 0.000
 NA's:205793   1st Qu.: 62.00   2: 95375   NA's:202659   1st Qu.: 4.013
                Median : 72.00                             Median :10.954
                Mean   : 71.51                             Mean   :15.844
                3rd Qu.: 81.00                             3rd Qu.:23.605
                Max.   :107.00                             Max.   :60.033
--------------------------------------------------------
dry_data[-which(dry_data$group == "c1" & dry_data[, "evero_bin"] == : 1
 dryamdonly    min_age      sex     death       survmon
 1    : 13    Min.   :50.00   1:74   1    :  7   Min.   : 0.000
 NA's:113    1st Qu.:53.00   2:52   NA's:119   1st Qu.: 5.115
             Median :57.00                      Median :13.372
```

```
           Mean   :57.83                          Mean   :19.772
           3rd Qu.:61.00                          3rd Qu.:32.694
           Max.   :73.00                          Max.   :59.868
```

```r
belat_bin_tab_dry #belatacept
```

```
dry_data[-which(dry_data$group == "c1" & dry_data[, "belat_bin"] == : 0
 dryamdonly       min_age        sex          death            survmon
 1   :  8123   Min.   : 50.00   1:118541   1    : 11257   Min.   : 0.000
 NA's:205793   1st Qu.: 62.00   2: 95375   NA's:202659   1st Qu.: 4.013
               Median : 72.00                            Median :10.954
               Mean   : 71.51                            Mean   :15.844
               3rd Qu.: 81.00                            3rd Qu.:23.605
               Max.   :107.00                            Max.   :60.033
--------------------------------------------------------
dry_data[-which(dry_data$group == "c1" & dry_data[, "belat_bin"] == : 1
 dryamdonly    min_age      sex    death      survmon
 1   :1     Min.   :53.0   1:4   1   :0   Min.   : 7.138
 NA's:4     1st Qu.:57.0   2:1   NA's:5   1st Qu.:10.612
            Median :58.0                  Median :32.763
            Mean   :61.4                  Mean   :30.978
            3rd Qu.:65.0                  3rd Qu.:46.546
            Max.   :74.0                  Max.   :57.829
```

```r
pred_bin_tab_dry #prednisone
```

```
dry_data[-which(dry_data$group == "c1" & dry_data[, "pred_bin"] == : 0
 dryamdonly       min_age        sex          death            survmon
 1   :  6925   Min.   : 50.00   1:101956   1    :  9575   Min.   : 0.00
 NA's:176425   1st Qu.: 62.00   2: 81394   NA's:173775   1st Qu.: 4.00
               Median : 72.00                            Median :10.84
               Mean   : 71.57                            Mean   :15.64
               3rd Qu.: 81.00                            3rd Qu.:23.31
               Max.   :107.00                            Max.   :60.03
--------------------------------------------------------
dry_data[-which(dry_data$group == "c1" & dry_data[, "pred_bin"] == : 1
 dryamdonly      min_age        sex         death          survmon
 1   : 1555   Min.   : 50.00   1:21773   1    : 2156   Min.   : 0.000
 NA's:37164   1st Qu.: 59.00   2:16946   NA's:36563   1st Qu.: 4.375
              Median : 68.00                           Median :11.967
```

```
Mean   : 68.69                          Mean   :17.501
3rd Qu.: 78.00                          3rd Qu.:26.483
Max.   :103.00                          Max.   :60.000
```

Cox Proportional Hazards Regression – Dry AMD

	Hazard Ratio	lower .95	upper .95	P-Value
Minimum Age	0.9975	0.9956	0.9993	0.0063

Table 1: Dry AMD Minimum Age

	Hazard Ratio	lower .95	upper .95	P-Value
<=70	0.9629	0.9230	1.0045	0.0796

Table 2: Dry AMD Median Age

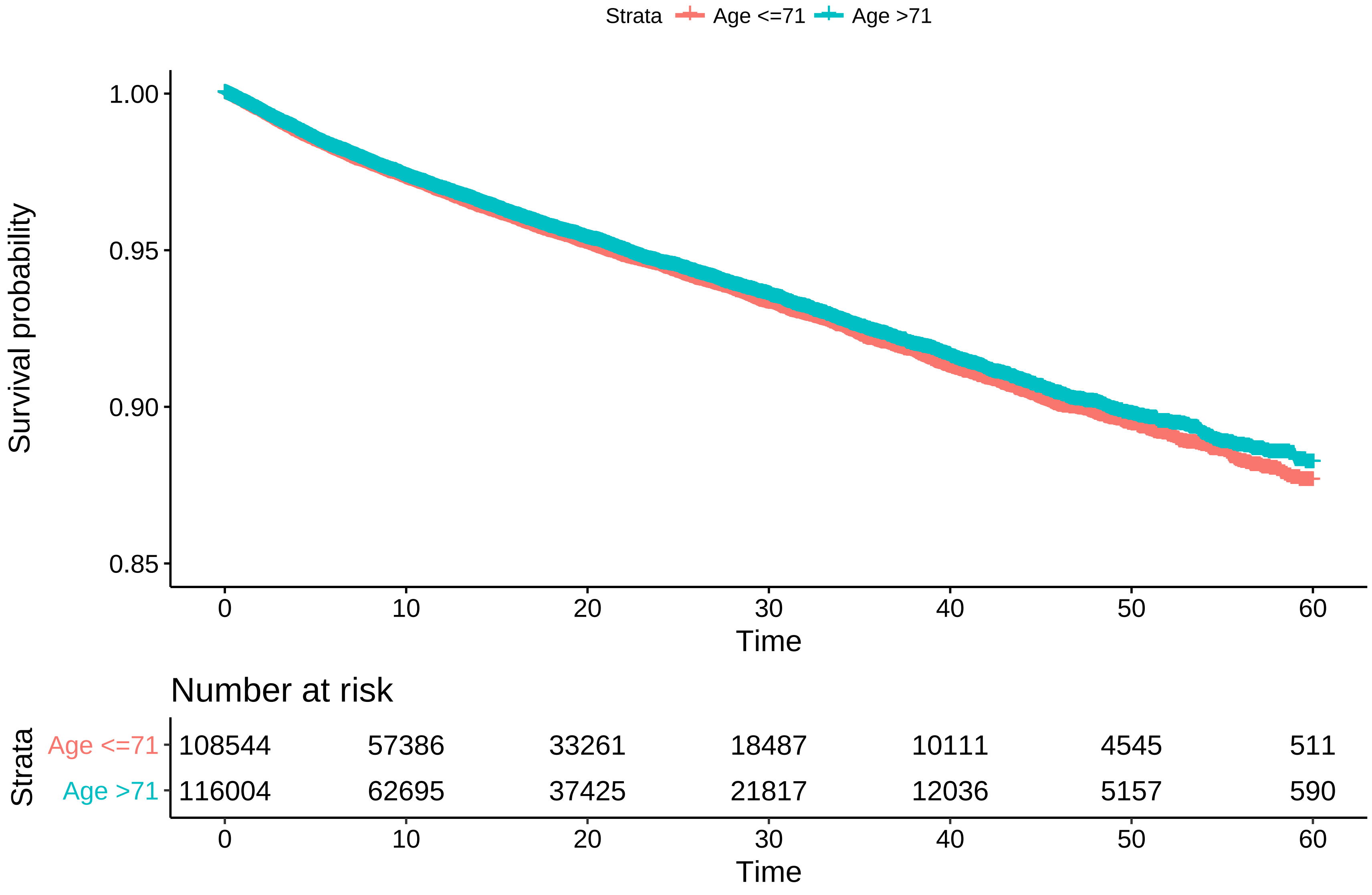
Strata
Age <=71
Age >71
Survival probability
1.00
0.95
0.90
0.85
0
10
20
30
40
50
60
Time
Number at risk
Strata
Age <=71
Age >71
108544 57386 33261 18487 10111 4545 511
116004 62695 37425 21817 12036 5157 590
Time

	Hazard Ratio	lower .95	upper .95	P-Value
Minimum Age	0.9971	0.9952	0.9990	0.0026
Everolimus:1	2.1428	1.2429	3.6942	0.0061

Table 3: Dry AMD Minimum Age and Everolimus

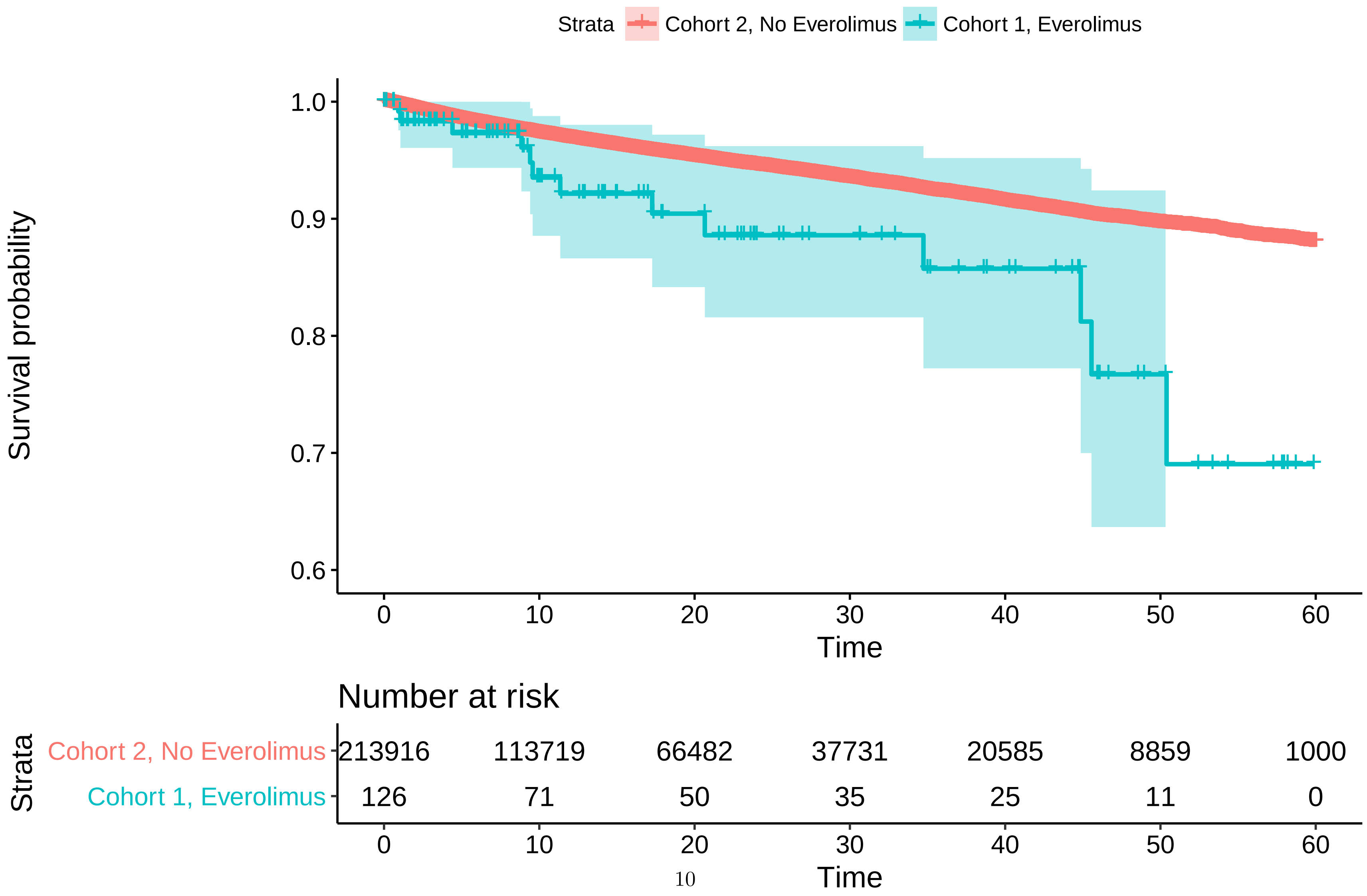

	Hazard Ratio	lower .95	upper .95	P-Value
Minimum Age	0.9971	0.9953	0.9990	0.0030
Sex:Female	1.0013	0.9583	1.0462	0.9528
Mycophenolate Mofetil:1	0.8175	0.6891	0.9697	0.0207
Sex:Female*Mycophenolate Mofetil:1	1.3713	1.0561	1.7805	0.0178

Table 4: Dry AMD Minimum Age and Mycophenolate Mofetil

```
Warning: Removed 25 rows containing missing values (geom_path).
```

```
Warning: Removed 25 rows containing missing values (geom_point).
```

```
Warning: Removed 25 rows containing missing values (geom_path).
```

```
Warning: Removed 25 rows containing missing values (geom_point).
```

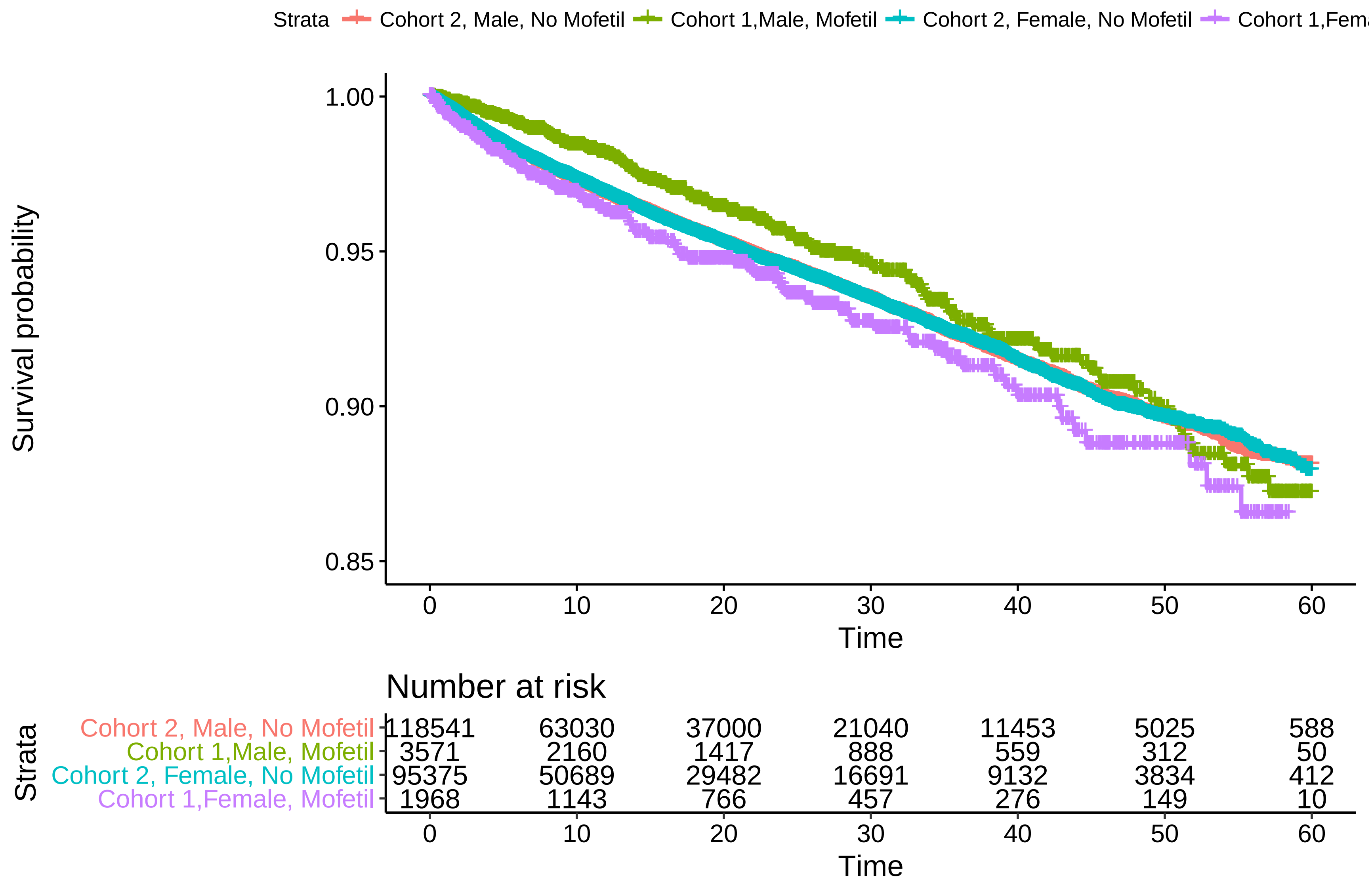
Strata
Cohort 2, Male, No Mofetil
Cohort 1,Male, Mofetil
Cohort 2, Female, No Mofetil
Cohort 1,Fem
Survival probability
1.00
0.95
0.90
0.85
0
10
20
30
40
50
60
Time
Number at risk
Strata
Cohort 2, Male, No Mofetil
Cohort 1,Male, Mofetil
Cohort 2, Female, No Mofetil
Cohort 1,Female, Mofetil
118541 63030 37000 21040 11453 5025 588
3571 2160 1417 888 559 312 50
95375 50689 29482 16691 9132 3834 412
1968 1143 766 457 276 149 10
0 10 20 30 40 50 60
Time

Wet AMD

For the wet amd group a basic table of sex, age, death, censor and survival time are displayed. And for each specific drug, tables are listed broken down by basic demographics. Cox proportional hazards regression model is used to assess the effects of age and each drug. *None of the drugs or age were significant.*

Simple Wet AMD Tables

Here a a list of simple tables related to Wet AMD

```
res_tab_wet #breakdown of wet amd. 1=Had Wet AMD only NA=Did not have Wet AMD
```

```
     1   NA's    Sum
  1505 215956 217461
```

```
sex_tab_wet #breakdown of wet amd by sex. 1=Women 2=Men
```

```
     1      2    Sum
121300  96161 217461
```

```
plot(age_hist_wet) #breakdown of wet amd: histogram of minimum age for each subject
```

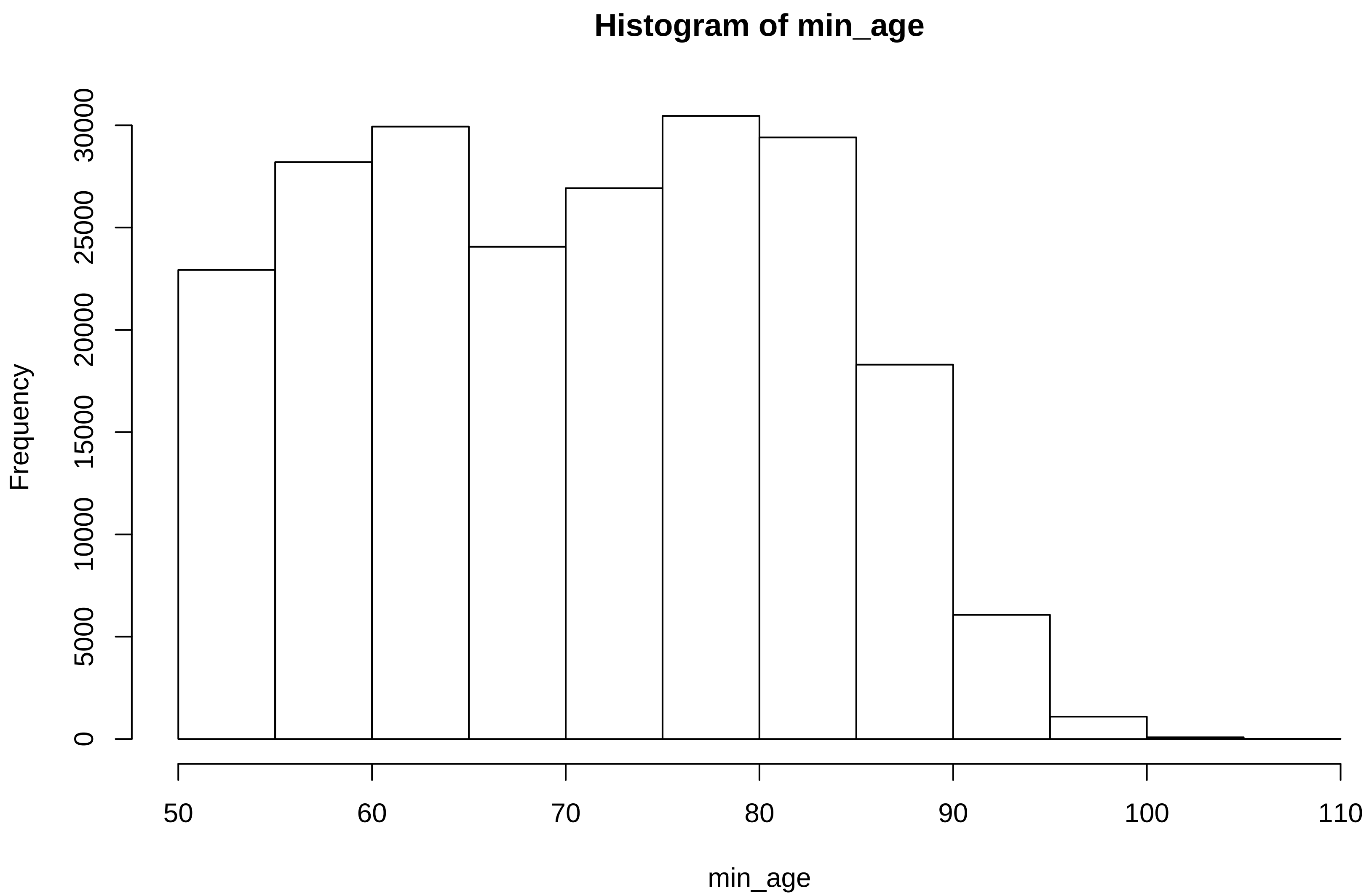


```
age_tab_wet #breakdown of wet amd: summary table of miniumum age
```

```
   Min. 1st Qu.  Median    Mean 3rd Qu.    Max. 
  50.00   61.00   71.00   70.94   81.00  107.00 
```

```
death_tab_wet #breakdown of wet amd: 1=Died; NA=No infomation about death
```

```
     1   NA's    Sum 
 11545 205916 217461 
```

```
censor_tab_wet #breakdown of wet amd: 1=Censored; 0=No Censored
```

```
     0      1    Sum 
  1505 215956 217461 
```

```
survmon_tab_wet #breakdown of wet amd: summary table of the survival months by subject
```

```
   Min. 1st Qu.  Median    Mean 3rd Qu.    Max. 
  0.000   4.053  11.000  16.058  23.829  60.033 
```

Wet AMD Tables by Drug

Here a a list of ables related to Wet AMD by drug.

```
#top part of tables are for those who were not on the drug (0)
#bottom part of tables are for those who were on the drug (1)
mycomo_bin_tab_wet # mycophenolate mofetil
```

```
wet_data[-which(wet_data$group == "c1" & wet_data[, "mycomo_bin"] == : 0
 eamdonly         min_age        sex          death           survmon     
 1    :  1435   Min.   : 50.00   1:114796   1    : 10948   Min.   : 0.00  
 NA's:205793   1st Qu.: 62.00   2: 92432   NA's:196280   1st Qu.: 4.02  
               Median : 72.00                            Median :10.97  
               Mean   : 71.51                            Mean   :15.91  
               3rd Qu.: 81.00                            3rd Qu.:23.66  
               Max.   :107.00                            Max.   :60.03  
------------------------------------------------------------------------ 
wet_data[-which(wet_data$group == "c1" & wet_data[, "mycomo_bin"] == : 1
 eamdonly       min_age       sex        death          survmon      
 1    :  46   Min.   :50.00   1:3463   1    : 324   Min.   : 0.000  
 NA's:5301   1st Qu.:54.00   2:1884   NA's:5023   1st Qu.: 4.921  
             Median :58.00                         Median :13.947  
             Mean   :59.13                         Mean   :19.146  
```

```
             3rd Qu.:63.00                                3rd Qu.:29.052
             Max.   :87.00                                Max.   :60.000
```

```r
metho_bin_tab_wet #methotrexate
```

```
wet_data[-which(wet_data$group == "c1" & wet_data[, "metho_bin"] == : 0
 eamdonly          min_age        sex          death            survmon
 1   :  1435   Min.   : 50.00   1:114796   1   : 10948   Min.   : 0.00
 NA's:205793   1st Qu.: 62.00   2: 92432   NA's:196280   1st Qu.: 4.02
               Median : 72.00                            Median :10.97
               Mean   : 71.51                            Mean   :15.91
               3rd Qu.: 81.00                            3rd Qu.:23.66
               Max.   :107.00                            Max.   :60.03
--------------------------------------------------------
wet_data[-which(wet_data$group == "c1" & wet_data[, "metho_bin"] == : 1
 eamdonly    min_age    sex    death      survmon
 1   :0   Min.   :52   1:2   1   :0   Min.   : 7.375
 NA's:3   1st Qu.:54   2:1   NA's:3   1st Qu.:30.529
          Median :56                  Median :53.684
          Mean   :62                  Mean   :40.353
          3rd Qu.:67                  3rd Qu.:56.842
          Max.   :78                  Max.   :60.000
```

```r
cyclo_bin_tab_wet #cyclosporine
```

```
wet_data[-which(wet_data$group == "c1" & wet_data[, "cyclo_bin"] == : 0
 eamdonly          min_age        sex          death            survmon
 1   :  1435   Min.   : 50.00   1:114796   1   : 10948   Min.   : 0.00
 NA's:205793   1st Qu.: 62.00   2: 92432   NA's:196280   1st Qu.: 4.02
               Median : 72.00                            Median :10.97
               Mean   : 71.51                            Mean   :15.91
               3rd Qu.: 81.00                            3rd Qu.:23.66
               Max.   :107.00                            Max.   :60.03
--------------------------------------------------------
wet_data[-which(wet_data$group == "c1" & wet_data[, "cyclo_bin"] == : 1
 eamdonly       min_age        sex       death        survmon
 1   :  7    Min.   :50.00   1:522   1   : 56    Min.   : 0.000
 NA's:828   1st Qu.:55.00   2:313   NA's:779    1st Qu.: 4.875
            Median :59.00                       Median :14.059
            Mean   :59.88                       Mean   :19.124
```

```
            3rd Qu.:63.00                          3rd Qu.:28.092  
            Max.   :86.00                          Max.   :60.000  
```

```r
tacro_bin_tab_wet #tacrolimus
```

```
wet_data[-which(wet_data$group == "c1" & wet_data[, "tacro_bin"] == : 0
 eamdonly          min_age         sex            death             survmon     
 1   :  1435   Min.   : 50.00   1:114796   1   : 10948   Min.   : 0.00  
 NA's:205793   1st Qu.: 62.00   2: 92432   NA's:196280   1st Qu.: 4.02  
               Median : 72.00                            Median :10.97  
               Mean   : 71.51                            Mean   :15.91  
               3rd Qu.: 81.00                            3rd Qu.:23.66  
               Max.   :107.00                            Max.   :60.03  
--------------------------------------------------------
wet_data[-which(wet_data$group == "c1" & wet_data[, "tacro_bin"] == : 1
 eamdonly        min_age       sex        death           survmon      
 1   :  44   Min.   :50.00   1:3767   1   : 364   Min.   : 0.000  
 NA's:5868   1st Qu.:54.00   2:2145   NA's:5548   1st Qu.: 4.865  
             Median :58.00                        Median :13.694  
             Mean   :58.89                        Mean   :19.036  
             3rd Qu.:62.00                        3rd Qu.:29.416  
             Max.   :89.00                        Max.   :60.000  
```

```r
siro_bin_tab_wet #sirolimus
```

```
wet_data[-which(wet_data$group == "c1" & wet_data[, "siro_bin"] == : 0
 eamdonly          min_age         sex            death             survmon     
 1   :  1435   Min.   : 50.00   1:114796   1   : 10948   Min.   : 0.00  
 NA's:205793   1st Qu.: 62.00   2: 92432   NA's:196280   1st Qu.: 4.02  
               Median : 72.00                            Median :10.97  
               Mean   : 71.51                            Mean   :15.91  
               3rd Qu.: 81.00                            3rd Qu.:23.66  
               Max.   :107.00                            Max.   :60.03  
--------------------------------------------------------
wet_data[-which(wet_data$group == "c1" & wet_data[, "siro_bin"] == : 1
 eamdonly       min_age        sex      death          survmon      
 1   :  1   Min.   :50.00   1:94   1   : 11   Min.   : 0.000  
 NA's:141   1st Qu.:52.25   2:48   NA's:131   1st Qu.: 4.408  
            Median :57.00                     Median :13.165  
            Mean   :57.73                     Mean   :19.348  
            3rd Qu.:61.00                     3rd Qu.:30.468  
```

```
           Max.   :76.00                       Max.   :60.000
```

```r
azath_bin_tab_wet #azathioprine
```

```
wet_data[-which(wet_data$group == "c1" & wet_data[, "azath_bin"] == : 0
 eamdonly          min_age        sex          death            survmon
 1   :  1435   Min.   : 50.00   1:114796   1    : 10948   Min.   : 0.00
 NA's:205793   1st Qu.: 62.00   2: 92432   NA's:196280   1st Qu.: 4.02
               Median : 72.00                            Median :10.97
               Mean   : 71.51                            Mean   :15.91
               3rd Qu.: 81.00                            3rd Qu.:23.66
               Max.   :107.00                            Max.   :60.03
--------------------------------------------------------
wet_data[-which(wet_data$group == "c1" & wet_data[, "azath_bin"] == : 1
 eamdonly      min_age       sex      death         survmon
 1   :  3   Min.   :50.00   1:181   1   : 21   Min.   : 0.000
 NA's:327   1st Qu.:53.00   2:149   NA's:309   1st Qu.: 7.946
            Median :57.00                      Median :18.421
            Mean   :58.38                      Mean   :21.249
            3rd Qu.:62.00                      3rd Qu.:32.469
            Max.   :83.00                      Max.   :59.967
```

```r
evero_bin_tab_wet #everolimus
```

```
wet_data[-which(wet_data$group == "c1" & wet_data[, "evero_bin"] == : 0
 eamdonly          min_age        sex          death            survmon
 1   :  1435   Min.   : 50.00   1:114796   1    : 10948   Min.   : 0.00
 NA's:205793   1st Qu.: 62.00   2: 92432   NA's:196280   1st Qu.: 4.02
               Median : 72.00                            Median :10.97
               Mean   : 71.51                            Mean   :15.91
               3rd Qu.: 81.00                            3rd Qu.:23.66
               Max.   :107.00                            Max.   :60.03
--------------------------------------------------------
wet_data[-which(wet_data$group == "c1" & wet_data[, "evero_bin"] == : 1
 eamdonly      min_age       sex     death         survmon
 1   :  2   Min.   :50.00   1:69   1   :  4   Min.   : 0.000
 NA's:113   1st Qu.:53.00   2:46   NA's:111   1st Qu.: 5.037
            Median :57.00                     Median :13.816
            Mean   :57.68                     Mean   :19.595
            3rd Qu.:60.50                     3rd Qu.:31.355
```

```
           Max.   :73.00                        Max.   :59.868
```

```r
belat_bin_tab_wet #belatacept
```

```
wet_data[-which(wet_data$group == "c1" & wet_data[, "belat_bin"] == : 0
 eamdonly          min_age        sex          death            survmon
 1   :  1435   Min.   : 50.00   1:114796   1    : 10948   Min.   : 0.00
 NA's:205793   1st Qu.: 62.00   2: 92432   NA's:196280   1st Qu.: 4.02
               Median : 72.00                            Median :10.97
               Mean   : 71.51                            Mean   :15.91
               3rd Qu.: 81.00                            3rd Qu.:23.66
               Max.   :107.00                            Max.   :60.03
--------------------------------------------------------
wet_data[-which(wet_data$group == "c1" & wet_data[, "belat_bin"] == : 1
 eamdonly    min_age       sex    death      survmon
 1   :0   Min.   :53.00   1:3   1   :0   Min.   : 7.138
 NA's:4   1st Qu.:56.00   2:1   NA's:4   1st Qu.: 9.743
          Median :57.50                  Median :28.579
          Mean   :58.25                  Mean   :30.531
          3rd Qu.:59.75                  3rd Qu.:49.367
          Max.   :65.00                  Max.   :57.829
```

```r
pred_bin_tab_wet #prednisone
```

```
wet_data[-which(wet_data$group == "c1" & wet_data[, "pred_bin"] == : 0
 eamdonly          min_age        sex         death            survmon
 1   :  1192   Min.   : 50.00   1:98737   1    :  9310   Min.   : 0.00
 NA's:176425   1st Qu.: 62.00   2:78880   NA's:168307   1st Qu.: 4.00
               Median : 72.00                           Median :10.88
               Mean   : 71.58                           Mean   :15.70
               3rd Qu.: 81.00                           3rd Qu.:23.41
               Max.   :107.00                           Max.   :60.03
--------------------------------------------------------
wet_data[-which(wet_data$group == "c1" & wet_data[, "pred_bin"] == : 1
 eamdonly         min_age        sex         death           survmon
 1   :  295   Min.   : 50.00   1:21060   1    : 2092   Min.   : 0.000
 NA's:37164   1st Qu.: 59.00   2:16399   NA's:35367   1st Qu.: 4.349
              Median : 68.00                          Median :11.967
              Mean   : 68.69                          Mean   :17.556
              3rd Qu.: 78.00                          3rd Qu.:26.651
```

```
                 Max.   :103.00                                 Max.   :60.000
```

Cox Proportional Hazards Regression – Wet AMD

Nothing to report here as none of the results were significant.

Dry to Wet AMD

For the dry to wet AMD group a basic table of sex, age, death, censor and survival time are displayed. And for each specific drug, tables are listed broken down by basic demographics. Cox proportional hazards regression model is used to assess the effects of age and each drug. A statistical interaction between minimum age and mycophenolate mofetil was found (HR Estimate=0.92, 95% CI: (0.85,0.99), p-value=0.0223). This result suggests that those who are older and on the drug have a smaller estimated hazard for developing Dry to Wet AMD compared to those who are older and not on the drug. While those who are younger and on the drug have a larger estimated hazard developing Dry to Wet AMD compared to those who are younger and not on the drug.

Simple Dry to Wet AMD Tables

Here a a list of simple tables related to Dry to Wet AMD.

```
res_tab_dtow #breakdown of dry to wet amd. 1=Had Dry AMD only NA=Did not have Dry AMD
```

```
     1   NA's    Sum
   939 215956 216895
```

```
sex_tab_dtow #breakdown of dry to wet amd by sex. 1=Women 2=Men
```

```
     1      2    Sum
121004  95891 216895
```

```
plot(age_hist_dtow) #breakdown of dry to wet amd: histogram of minimum age for each subject
```

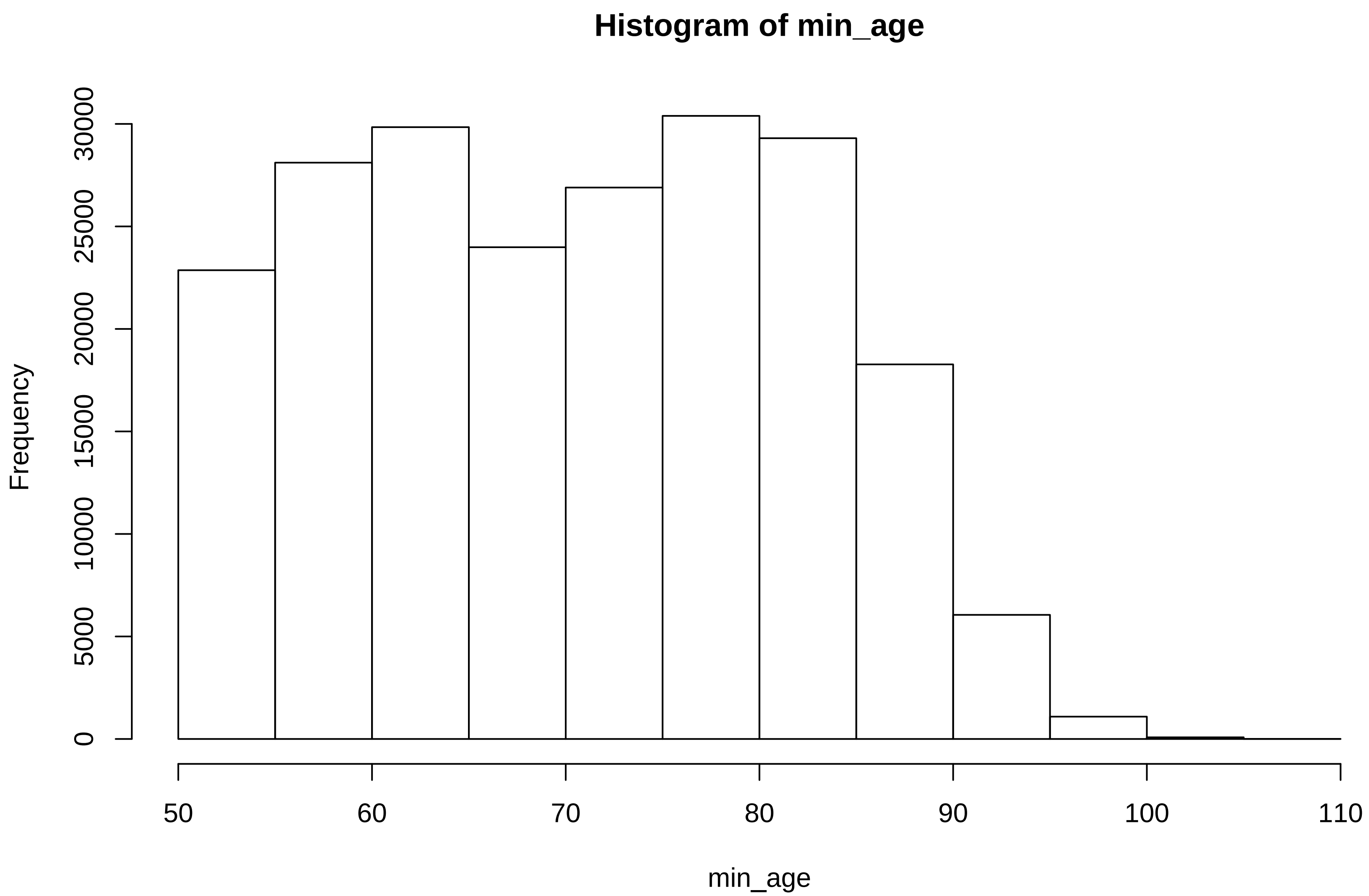


```
age_tab_dtow #breakdown of dry to wet amd: summary table of miniumum age
```

```
   Min. 1st Qu.  Median    Mean 3rd Qu.    Max. 
  50.00   61.00   71.00   70.95   81.00  107.00 
```

```r
death_tab_dtow #breakdown of dry to wet amd: 1=Died; NA=No infomation about death
```

```
     1   NA's    Sum 
 11488 205407 216895 
```

```r
censor_tab_dtow #breakdown of dry to wet amd: 1=Censored; 0=No Censored
```

```
     0      1    Sum 
   939 215956 216895 
```

```r
survmon_tab_dtow #breakdown of dry to wet amd: summary table of the survival months by subject
```

```
   Min. 1st Qu.  Median    Mean 3rd Qu.    Max. 
  0.000   4.079  11.033  16.097  23.855  60.033 
```

There were 939 subjects who had dry AMD and were in cohort 1.

Dry to wet AMD Tables by Drug

Here a a list of ables related to Dry to wet AMD by drug.

```r
#top part of tables are for those who were not on the drug (0)
#bottom part of tables are for those who were on the drug (1)
mycomo_bin_tab_dtow # mycophenolate mofetil
```

```
dtow_data[, "mycomo_bin"]: 0
 drytowet          min_age        sex          death            survmon      
 1   :   912   Min.   : 50.00   1:117555   1   : 11164   Min.   : 0.000  
 NA's:210655   1st Qu.: 61.00   2: 94012   NA's:200403   1st Qu.: 4.059  
               Median : 72.00                            Median :11.000  
               Mean   : 71.25                            Mean   :16.019  
               3rd Qu.: 81.00                            3rd Qu.:23.770  
               Max.   :107.00                            Max.   :60.033  
--------------------------------------------------------
dtow_data[, "mycomo_bin"]: 1
 drytowet        min_age        sex        death         survmon      
 1   :  27   Min.   :50.00   1:3449   1   : 324   Min.   : 0.000  
 NA's:5301   1st Qu.:54.00   2:1879   NA's:5004   1st Qu.: 4.934  
             Median :58.00                        Median :14.000  
```

```
             Mean   :59.13                               Mean   :19.209
             3rd Qu.:63.00                               3rd Qu.:29.198
             Max.   :87.00                               Max.   :60.000
```

```r
metho_bin_tab_dtow #methotrexate
```

```
dtow_data[, "metho_bin"]: 0
 drytowet          min_age        sex          death            survmon
 1    :   939   Min.   : 50.00   1:121002   1    : 11488   Min.   : 0.000
 NA's:215953   1st Qu.: 61.00   2: 95890   NA's:205404   1st Qu.: 4.079
               Median : 71.00                            Median :11.033
               Mean   : 70.95                            Mean   :16.097
               3rd Qu.: 81.00                            3rd Qu.:23.855
               Max.   :107.00                            Max.   :60.033
--------------------------------------------------------
dtow_data[, "metho_bin"]: 1
 drytowet    min_age    sex    death      survmon
 1    :0   Min.   :52   1:2   1    :0   Min.   : 7.375
 NA's:3    1st Qu.:54   2:1   NA's:3   1st Qu.:30.529
           Median :56                  Median :53.684
           Mean   :62                  Mean   :40.353
           3rd Qu.:67                  3rd Qu.:56.842
           Max.   :78                  Max.   :60.000
```

```r
cyclo_bin_tab_dtow #cyclosporine
```

```
dtow_data[, "cyclo_bin"]: 0
 drytowet          min_age        sex          death            survmon
 1    :   936   Min.   : 50.00   1:120484   1    : 11432   Min.   : 0.000
 NA's:215128   1st Qu.: 61.00   2: 95580   NA's:204632   1st Qu.: 4.079
               Median : 71.00                            Median :11.020
               Mean   : 70.99                            Mean   :16.086
               3rd Qu.: 81.00                            3rd Qu.:23.849
               Max.   :107.00                            Max.   :60.033
--------------------------------------------------------
dtow_data[, "cyclo_bin"]: 1
 drytowet      min_age       sex      death        survmon
 1    :  3   Min.   :50.00   1:520   1    : 56   Min.   : 0.000
 NA's:828   1st Qu.:54.50   2:311   NA's:775   1st Qu.: 4.875
            Median :59.00                      Median :14.059
```

```
             Mean   :59.89                           Mean   :19.173
             3rd Qu.:63.00                           3rd Qu.:28.122
             Max.   :86.00                           Max.   :60.000
```

```
tacro_bin_tab_dtow #tacrolimus
```

```
dtow_data[, "tacro_bin"]: 0
 drytowet         min_age        sex          death            survmon
 1   :   903   Min.   : 50.00   1:117247   1   : 11124   Min.   : 0.000
 NA's:210088   1st Qu.: 61.00   2: 93744   NA's:199867   1st Qu.: 4.059
               Median : 72.00                            Median :11.000
               Mean   : 71.29                            Mean   :16.014
               3rd Qu.: 81.00                            3rd Qu.:23.750
               Max.   :107.00                            Max.   :60.033
--------------------------------------------------------
dtow_data[, "tacro_bin"]: 1
 drytowet       min_age       sex       death          survmon
 1   :  36   Min.   :50.00   1:3757   1   : 364   Min.   : 0.000
 NA's:5868   1st Qu.:54.00   2:2147   NA's:5540   1st Qu.: 4.901
             Median :58.00                        Median :13.822
             Mean   :58.88                        Mean   :19.081
             3rd Qu.:62.00                        3rd Qu.:29.476
             Max.   :89.00                        Max.   :60.000
```

```
siro_bin_tab_dtow #sirolimus
```

```
dtow_data[, "siro_bin"]: 0
 drytowet         min_age        sex          death            survmon
 1   :   938   Min.   : 50.00   1:120910   1   : 11477   Min.   : 0.000
 NA's:215815   1st Qu.: 61.00   2: 95843   NA's:205276   1st Qu.: 4.079
               Median : 71.00                            Median :11.026
               Mean   : 70.96                            Mean   :16.095
               3rd Qu.: 81.00                            3rd Qu.:23.855
               Max.   :107.00                            Max.   :60.033
--------------------------------------------------------
dtow_data[, "siro_bin"]: 1
 drytowet      min_age       sex     death         survmon
 1   :  1   Min.   :50.00   1:94   1   : 11   Min.   : 0.000
 NA's:141   1st Qu.:52.25   2:48   NA's:131   1st Qu.: 4.408
            Median :57.00                     Median :13.671
            Mean   :57.74                     Mean   :19.539
```

```
           3rd Qu.:61.00                     3rd Qu.:31.148
           Max.   :76.00                     Max.   :60.000
```

```
azath_bin_tab_dtow #azathioprine
```

```
dtow_data[, "azath_bin"]: 0
 drytowet         min_age         sex          death            survmon
 1   :   938   Min.   : 50.00   1:120823   1   : 11467   Min.   : 0.000
 NA's:215629   1st Qu.: 61.00   2: 95744   NA's:205100   1st Qu.: 4.079
               Median : 71.00                            Median :11.020
               Mean   : 70.97                            Mean   :16.089
               3rd Qu.: 81.00                            3rd Qu.:23.849
               Max.   :107.00                            Max.   :60.033
--------------------------------------------------------
dtow_data[, "azath_bin"]: 1
 drytowet      min_age       sex      death         survmon
 1   :  1   Min.   :50.00   1:181   1   : 21   Min.   : 0.000
 NA's:327   1st Qu.:53.00   2:147   NA's:307   1st Qu.: 7.982
            Median :57.00                      Median :18.421
            Mean   :58.37                      Mean   :21.375
            3rd Qu.:62.00                      3rd Qu.:33.341
            Max.   :83.00                      Max.   :59.967
```

```
evero_bin_tab_dtow #everolimus
```

```
dtow_data[, "evero_bin"]: 0
 drytowet         min_age         sex          death            survmon
 1   :   939   Min.   : 50.00   1:120937   1   : 11484   Min.   : 0.000
 NA's:215843   1st Qu.: 61.00   2: 95845   NA's:205298   1st Qu.: 4.079
               Median : 71.00                            Median :11.030
               Mean   : 70.96                            Mean   :16.095
               3rd Qu.: 81.00                            3rd Qu.:23.855
               Max.   :107.00                            Max.   :60.033
--------------------------------------------------------
dtow_data[, "evero_bin"]: 1
 drytowet      min_age       sex     death         survmon
 1   :  0   Min.   :50.00   1:67   1   :  4   Min.   : 0.000
 NA's:113   1st Qu.:53.00   2:46   NA's:109   1st Qu.: 5.066
            Median :57.00                     Median :13.816
            Mean   :57.69                     Mean   :19.754
```

```
           3rd Qu.:61.00                  3rd Qu.:32.053
           Max.   :73.00                  Max.   :59.868
```

```r
belat_bin_tab_dtow #belatacept
```

```
dtow_data[, "belat_bin"]: 0
 drytowet         min_age         sex           death            survmon
 1   :   939   Min.   : 50.00   1:121001   1   : 11488   Min.   : 0.000
 NA's:215952   1st Qu.: 61.00   2: 95890   NA's:205403   1st Qu.: 4.079
               Median : 71.00                            Median :11.033
               Mean   : 70.95                            Mean   :16.097
               3rd Qu.: 81.00                            3rd Qu.:23.855
               Max.   :107.00                            Max.   :60.033
--------------------------------------------------------
dtow_data[, "belat_bin"]: 1
 drytowet    min_age       sex    death      survmon
 1   :0   Min.   :53.00   1:3   1   :0   Min.   : 7.138
 NA's:4   1st Qu.:56.00   2:1   NA's:4   1st Qu.: 9.743
          Median :57.50                  Median :28.579
          Mean   :58.25                  Mean   :30.531
          3rd Qu.:59.75                  3rd Qu.:49.367
          Max.   :65.00                  Max.   :57.829
```

```r
pred_bin_tab_dtow #prednisone
```

```
dtow_data[, "pred_bin"]: 0
 drytowet         min_age         sex           death            survmon
 1   :   758   Min.   : 50.00   1:100007   1   :  9398   Min.   : 0.000
 NA's:178792   1st Qu.: 61.00   2: 79543   NA's:170152   1st Qu.: 4.033
               Median : 72.00                            Median :10.941
               Mean   : 71.42                            Mean   :15.787
               3rd Qu.: 81.00                            3rd Qu.:23.487
               Max.   :107.00                            Max.   :60.033
--------------------------------------------------------
dtow_data[, "pred_bin"]: 1
 drytowet        min_age         sex          death          survmon
 1   :  181   Min.   : 50.00   1:20997   1   : 2090   Min.   : 0.000
 NA's:37164   1st Qu.: 59.00   2:16348   NA's:35255   1st Qu.: 4.375
              Median : 68.00                          Median :11.967
              Mean   : 68.69                          Mean   :17.590
```

```
3rd Qu.: 78.00                          3rd Qu.:26.711
Max.   :103.00                          Max.   :60.000
```

Cox Proportional Hazards Regression – Dry to Wet AMD

Here an interaction between mycophenolate mofetil and minimum age was found. Below is a table of the

	Hazard Ratio	lower .95	upper .95	P-Value
Minimum Age	1.0046	0.9988	1.0105	0.1178
Mycophenolate mofetil:1	147.0717	2.2538	9597.2479	0.0192
Minimum Age*Mycophenolate Mofetil:1	0.9177	0.8526	0.9879	0.0223

Table 5: Dry to Wet AMD Interaction between Minimum Age and Mycophenolate Mofetil

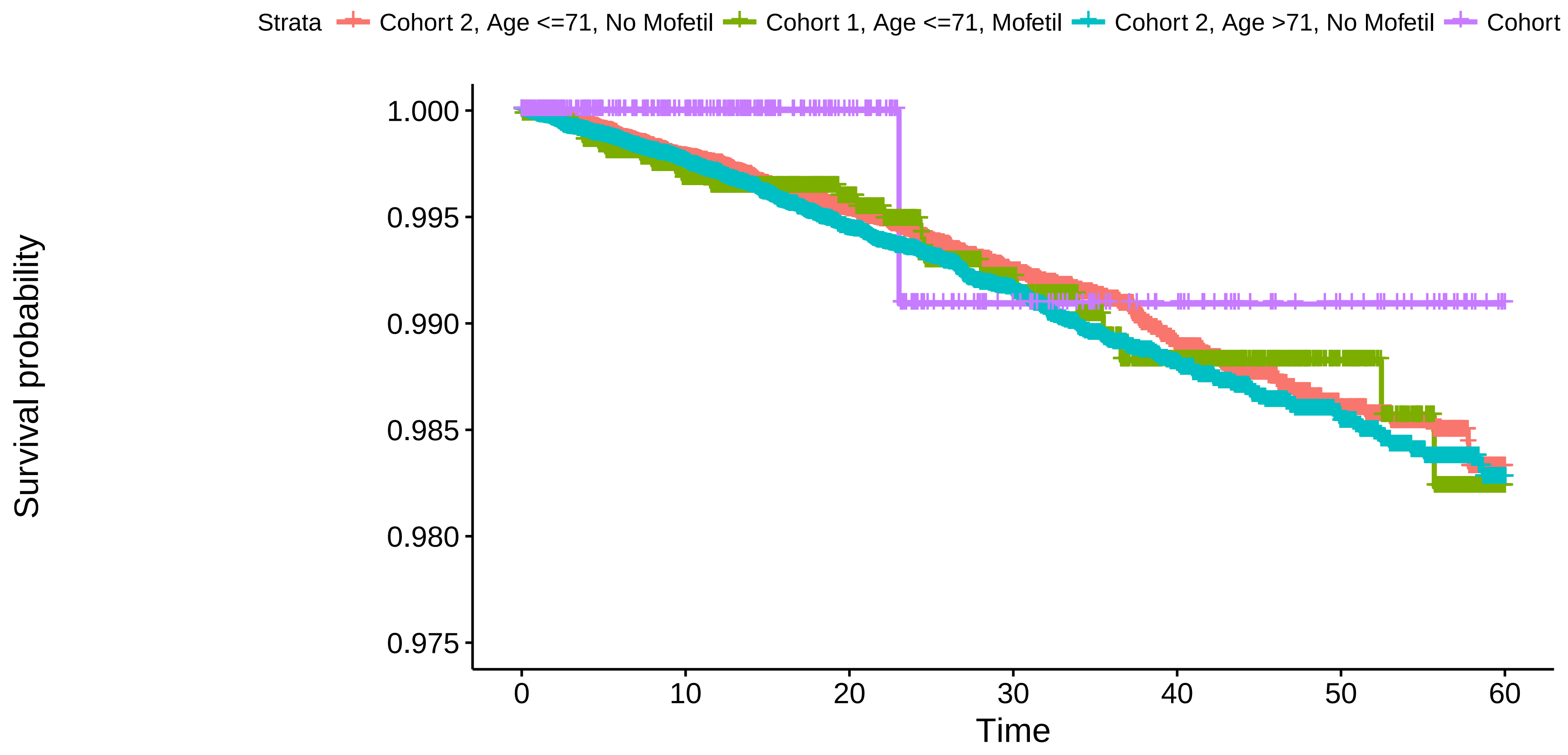

Number at risk

Strata	0	10	20	30	40	50	60
Cohort 2, Age <=71, No Mofetil	95330	50013	28617	15702	8492	3706	416
Cohort 1, Age <=71, Mofetil	4967	2970	1979	1222	764	426	58
Cohort 2, Age >71, No Mofetil	111340	60465	36175	21155	11733	5061	584
Cohort 1, Age >71, Mofetil	361	201	128	75	47	25	2

Time

NOS AMD

For the dry amd group a basic table of sex, age, death, censor and survival time are displayed. And for each specific drug, tables are listed broken down by basic demographics. Cox proportional hazards regression model is used to assess the effects of age and each drug. None of the drugs or age were found to be significant.

Simple NOS AMD Tables

Here a a list of simple tables related to NOS AMD.

```
res_tab_nos #breakdown of nos amd. 1=Had nos NA=Did not have nos
```

```
     1   NA's    Sum
   902 215956 216858
```

```
sex_tab_nos #breakdown of nos amd by sex. 1=Women 2=Men
```

```
     1      2    Sum
120992  95866 216858
```

```
plot(age_hist_nos) #breakdown of nos amd: histogram of minimum age for each subject
```

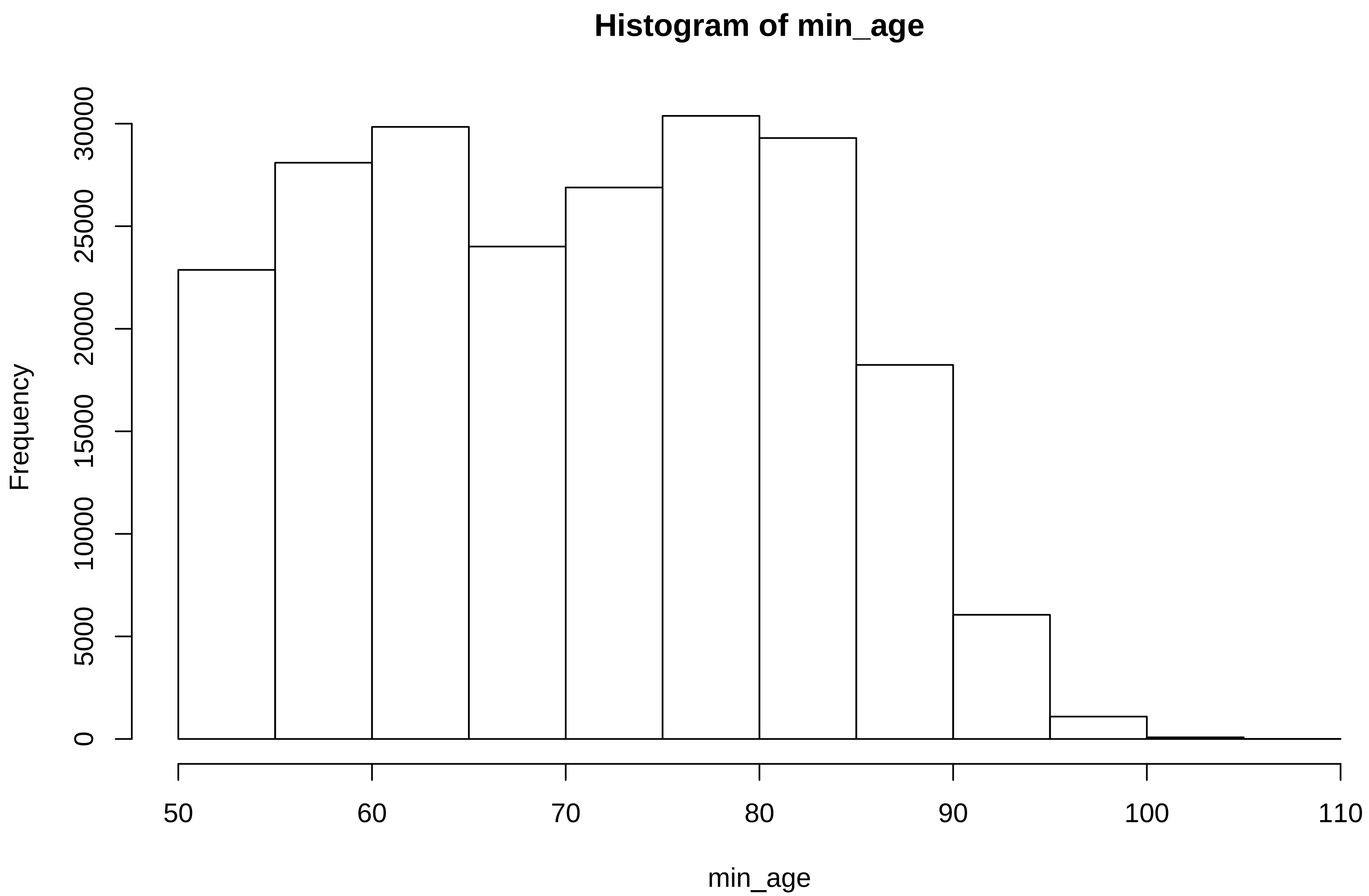


```
age_tab_nos #breakdown of nos amd: summary table of miniumum age
```

```
   Min. 1st Qu.  Median    Mean 3rd Qu.    Max. 
  50.00   61.00   71.00   70.95   81.00  107.00 
```

```r
death_tab_nos #breakdown of nos amd: 1=Died; NA=No infomation about death
```

```
     1   NA's    Sum 
 11490 205368 216858 
```

```r
censor_tab_nos #breakdown of nos amd: 1=Censored; 0=No Censored
```

```
     0      1    Sum 
   902 215956 216858 
```

```r
survmon_tab_nos #breakdown of nos amd: summary table of the survival months by subject
```

```
   Min. 1st Qu.  Median    Mean 3rd Qu.    Max. 
  0.000   4.059  11.000  16.072  23.836  60.033 
```

NOS AMD Tables by Drug

Here a a list of tables related to NOS AMD by drug.

```r
#top part of tables are for those who were not on the drug (0)
#bottom part of tables are for those who were on the drug (1)
mycomo_bin_tab_nos # mycophenolate mofetil
```

```
nos_data[, "mycomo_bin"]: 0
   nos             min_age        sex          death           survmon      
 1   :   876   Min.   : 50.00   1:117539   1   : 11166   Min.   : 0.000  
 NA's:210655   1st Qu.: 61.00   2: 93992   NA's:200365   1st Qu.: 4.039  
               Median : 72.00                            Median :11.000  
               Mean   : 71.24                            Mean   :15.994  
               3rd Qu.: 81.00                            3rd Qu.:23.750  
               Max.   :107.00                            Max.   :60.033  
-------------------------------------------------------- 
nos_data[, "mycomo_bin"]: 1
   nos           min_age      sex        death          survmon      
 1   :  26   Min.   :50.00   1:3453   1   : 324   Min.   : 0.000  
 NA's:5301   1st Qu.:54.00   2:1874   NA's:5003   1st Qu.: 4.921  
             Median :58.00                        Median :13.961  
             Mean   :59.14                        Mean   :19.175  
```

```
             3rd Qu.:63.00                      3rd Qu.:29.162  
             Max.   :87.00                      Max.   :60.000  
```

```r
metho_bin_tab_nos #methotrexate
```

```
nos_data[, "metho_bin"]: 0
   nos             min_age          sex           death             survmon      
 1   :   902   Min.   : 50.00   1:120990   1   : 11490   Min.   : 0.000  
 NA's:215953   1st Qu.: 61.00   2: 95865   NA's:205365   1st Qu.: 4.059  
               Median : 71.00                            Median :11.000  
               Mean   : 70.95                            Mean   :16.072  
               3rd Qu.: 81.00                            3rd Qu.:23.836  
               Max.   :107.00                            Max.   :60.033  
--------------------------------------------------------
nos_data[, "metho_bin"]: 1
   nos        min_age     sex     death       survmon      
 1   :0   Min.   :52   1:2   1   :0   Min.   : 7.375  
 NA's:3   1st Qu.:54   2:1   NA's:3   1st Qu.:30.529  
          Median :56                  Median :53.684  
          Mean   :62                  Mean   :40.353  
          3rd Qu.:67                  3rd Qu.:56.842  
          Max.   :78                  Max.   :60.000  
```

```r
cyclo_bin_tab_nos #cyclosporine
```

```
nos_data[, "cyclo_bin"]: 0
   nos             min_age          sex           death             survmon      
 1   :   899   Min.   : 50.00   1:120471   1   : 11434   Min.   : 0.000  
 NA's:215128   1st Qu.: 61.00   2: 95556   NA's:204593   1st Qu.: 4.053  
               Median : 71.00                            Median :11.000  
               Mean   : 70.99                            Mean   :16.060  
               3rd Qu.: 81.00                            3rd Qu.:23.836  
               Max.   :107.00                            Max.   :60.033  
--------------------------------------------------------
nos_data[, "cyclo_bin"]: 1
   nos           min_age        sex       death         survmon      
 1   :  3   Min.   :50.00   1:521   1   : 56   Min.   : 0.000  
 NA's:828   1st Qu.:54.50   2:310   NA's:775   1st Qu.: 4.875  
            Median :59.00                      Median :14.059  
            Mean   :59.89                      Mean   :19.184  
```

```
            3rd Qu.:63.00                      3rd Qu.:28.122
            Max.   :86.00                      Max.   :60.000
```

```r
tacro_bin_tab_nos #tacrolimus
```

```
nos_data[, "tacro_bin"]: 0
   nos             min_age        sex          death            survmon
 1   :    871   Min.   : 50.00   1:117233   1    : 11127   Min.   : 0.000
 NA's:210088   1st Qu.: 61.00   2: 93726   NA's:199832   1st Qu.: 4.039
               Median : 72.00                            Median :11.000
               Mean   : 71.28                            Mean   :15.988
               3rd Qu.: 81.00                            3rd Qu.:23.737
               Max.   :107.00                            Max.   :60.033
--------------------------------------------------------
nos_data[, "tacro_bin"]: 1
   nos           min_age      sex       death         survmon
 1   :  31    Min.   :50.00   1:3759   1    : 363   Min.   : 0.000
 NA's:5868   1st Qu.:54.00   2:2140   NA's:5536   1st Qu.: 4.868
             Median :58.00                         Median :13.717
             Mean   :58.89                         Mean   :19.060
             3rd Qu.:62.00                         3rd Qu.:29.464
             Max.   :89.00                         Max.   :60.000
```

```r
siro_bin_tab_nos #sirolimus
```

```
nos_data[, "siro_bin"]: 0
   nos             min_age        sex          death            survmon
 1   :    902   Min.   : 50.00   1:120899   1    : 11479   Min.   : 0.000
 NA's:215815   1st Qu.: 61.00   2: 95818   NA's:205238   1st Qu.: 4.059
               Median : 71.00                            Median :11.000
               Mean   : 70.95                            Mean   :16.070
               3rd Qu.: 81.00                            3rd Qu.:23.836
               Max.   :107.00                            Max.   :60.033
--------------------------------------------------------
nos_data[, "siro_bin"]: 1
   nos          min_age      sex     death        survmon
 1   :  0    Min.   :50.00   1:93   1    : 11   Min.   : 0.000
 NA's:141   1st Qu.:53.00   2:48   NA's:130   1st Qu.: 4.296
            Median :57.00                      Median :13.296
            Mean   :57.78                      Mean   :19.419
            3rd Qu.:61.00                      3rd Qu.:30.822
```

```
           Max.   :76.00                        Max.   :60.000
```

```r
azath_bin_tab_nos #azathioprine
```

```
nos_data[, "azath_bin"]: 0
   nos             min_age        sex          death            survmon
 1   :   900   Min.   : 50.00   1:120811   1    : 11469   Min.   : 0.000
 NA's:215629   1st Qu.: 61.00   2: 95718   NA's:205060   1st Qu.: 4.053
               Median : 71.00                            Median :11.000
               Mean   : 70.96                            Mean   :16.064
               3rd Qu.: 81.00                            3rd Qu.:23.836
               Max.   :107.00                            Max.   :60.033
--------------------------------------------------------
nos_data[, "azath_bin"]: 1
   nos          min_age       sex      death         survmon
 1   :  2   Min.   :50.00   1:181   1   : 21   Min.   : 0.000
 NA's:327   1st Qu.:53.00   2:148   NA's:308   1st Qu.: 7.928
            Median :57.00                      Median :18.421
            Mean   :58.33                      Mean   :21.321
            3rd Qu.:62.00                      3rd Qu.:33.336
            Max.   :83.00                      Max.   :59.967
```

```r
evero_bin_tab_nos #everolimus
```

```
nos_data[, "evero_bin"]: 0
   nos             min_age        sex          death            survmon
 1   :   902   Min.   : 50.00   1:120925   1    : 11486   Min.   : 0.000
 NA's:215843   1st Qu.: 61.00   2: 95820   NA's:205259   1st Qu.: 4.059
               Median : 71.00                            Median :11.000
               Mean   : 70.95                            Mean   :16.070
               3rd Qu.: 81.00                            3rd Qu.:23.836
               Max.   :107.00                            Max.   :60.033
--------------------------------------------------------
nos_data[, "evero_bin"]: 1
   nos          min_age       sex     death        survmon
 1   :  0   Min.   :50.00   1:67   1   :  4   Min.   : 0.000
 NA's:113   1st Qu.:53.00   2:46   NA's:109   1st Qu.: 5.066
            Median :57.00                     Median :13.816
            Mean   :57.69                     Mean   :19.754
            3rd Qu.:61.00                     3rd Qu.:32.053
```

```
            Max.   :73.00                    Max.   :59.868
```

```r
belat_bin_tab_nos #belatacept
```

```
nos_data[, "belat_bin"]: 0
   nos             min_age        sex          death           survmon
 1    :    902   Min.   : 50.00   1:120989   1    : 11490   Min.   : 0.000
 NA's:215952   1st Qu.: 61.00   2: 95865   NA's:205364   1st Qu.: 4.059
               Median : 71.00                             Median :11.000
               Mean   : 70.95                             Mean   :16.072
               3rd Qu.: 81.00                             3rd Qu.:23.836
               Max.   :107.00                             Max.   :60.033
--------------------------------------------------------
nos_data[, "belat_bin"]: 1
   nos       min_age      sex    death       survmon
 1   :0   Min.   :53.00   1:3   1   :0   Min.   : 7.138
 NA's:4   1st Qu.:56.00   2:1   NA's:4   1st Qu.: 9.743
          Median :57.50                  Median :28.579
          Mean   :58.25                  Mean   :30.531
          3rd Qu.:59.75                  3rd Qu.:49.367
          Max.   :65.00                  Max.   :57.829
```

```r
pred_bin_tab_nos #prednisone
```

```
nos_data[, "pred_bin"]: 0
   nos             min_age        sex          death           survmon
 1    :    753   Min.   : 50.00   1:100011   1    :  9406   Min.   : 0.000
 NA's:178792   1st Qu.: 61.00   2: 79534   NA's:170139   1st Qu.: 4.007
               Median : 72.00                             Median :10.921
               Mean   : 71.42                             Mean   :15.760
               3rd Qu.: 81.00                             3rd Qu.:23.454
               Max.   :107.00                             Max.   :60.033
--------------------------------------------------------
nos_data[, "pred_bin"]: 1
   nos            min_age       sex         death          survmon
 1    :  149   Min.   : 50.00   1:20981   1    : 2084   Min.   : 0.000
 NA's:37164   1st Qu.: 59.00   2:16332   NA's:35229   1st Qu.: 4.349
              Median : 68.00                          Median :11.967
              Mean   : 68.68                          Mean   :17.572
              3rd Qu.: 78.00                          3rd Qu.:26.678
```

```
 Max.   :103.00                        Max.   :60.000
```

Cox Proportional Hazards Regression – NOS AMD

None of the drugs or age were found to be significant.
